# Measurement of benefits in economic evaluations of nutrition interventions in low‐ and middle‐income countries: A systematic review

**DOI:** 10.1111/mcn.13323

**Published:** 2022-02-08

**Authors:** Jolene Wun, Christopher Kemp, Chloe Puett, Devon Bushnell, Jonny Crocker, Carol Levin

**Affiliations:** ^1^ Independent Consultant Washington District of Columbia USA; ^2^ Department of Global Health University of Washington Seattle Washington USA; ^3^ Program in Public Health Stony Brook University Stony Brook New York USA

**Keywords:** cost‐benefit analysis, cost‐effectiveness analysis, economic evaluation, intervention, low‐ and middle‐income countries, malnutrition

## Abstract

Economic evaluation of nutrition interventions that compares the costs to benefits is essential to priority‐setting. However, there are unique challenges to synthesizing the findings of multi‐sectoral nutrition interventions due to the diversity of potential benefits and the methodological differences among sectors in measuring them. This systematic review summarises literature on the interventions, sectors, benefit terminology and benefit types included in cost‐effectiveness, cost‐utility and benefit‐cost analyses (CEA, CUA and BCA, respectively) of nutrition interventions in low‐ and middle‐income countries. A systematic search of five databases published from January 2010 to September 2019 with expert consultation yielded 2794 studies, of which 93 met all inclusion criteria. Eighty‐seven per cent of the included studies included interventions delivered from only one sector, with almost half from the health sector (43%), followed by food/agriculture (27%), water, sanitation and hygiene (WASH) (10%), and social protection (8%). Only 9% of studies assessed programmes involving more than one sector (health, food/agriculture, social protection and/or WASH). Eighty‐one per cent of studies used more than one term to refer to intervention benefits. The included studies calculated 128 economic evaluation ratios (57 CEAs, 39 CUAs and 32 BCAs), and the benefits they included varied by sector. Nearly 60% measured a single benefit category, most frequently nutritional status improvements; other health benefits, cognitive/education gains, dietary diversity, food security, knowledge/attitudes/practices and income were included in less than 10% of all ratios. Additional economic evaluation of non‐health and multi‐sector interventions, and incorporation of benefits beyond nutritional improvements (including cost savings) in future economic evaluations is recommended.

## Key messages

- Current economic evaluations often underestimate the total sum of benefits that can arise from nutrition interventions. Comprehensive benefit measurement of some nutrition programmes may require further methodological research.
- In the near‐term, economic evaluations of multi‐sectoral nutrition interventions should include potential cost savings from improved nutrition in their calculations and assess the potential for benefits unrelated to nutrition. If the range of benefits is diverse and can be monetised, benefit‐cost analysis may be the preferred evaluation method.
- Economic evaluations of nutrition‐sensitive interventions from agriculture, water, sanitation and hygiene (WASH), and gender empowerment sectors, are needed to fill an evidence gap on costs and benefits of multisectoral approaches to improved maternal and child health and nutrition.

## Graphical abstract

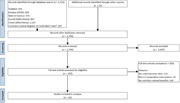

## 1 INTRODUCTION

Malnutrition is widely acknowledged by governments, international agencies, donors and researchers as a problem with diverse causes, requiring multiple strategies and the engagement of multiple sectors. Sixty‐one country governments are part of the United Nations (UN) Network for Scaling Up Nutrition (SUN) Movement, which explicitly calls for a multi‐stakeholder and multi‐sectoral approach to improving nutrition outcomes (Scaling Up Nutrition, 2020). This approach combines both *nutrition‐specific* interventions (those that address the immediate causes of malnutrition, such as micronutrient supplementation, treatment of acute malnutrition and promotion of appropriate dietary and feeding behaviours) and *nutrition‐sensitive* interventions (those that address the underlying causes, such as ensuring child protection, women's empowerment, agricultural production and adequate water and sanitation; Ruel et al., 2013). To assist countries in developing multi‐sectoral nutrition strategies, SUN and the UN Renewed Efforts Against Child Hunger (REACH) initiative published the *Compendium of Actions for Nutrition*, a wide‐ranging menu of nutrition‐specific and nutrition‐sensitive interventions (World Food Programme, 2016).

Given the diversity of nutrition‐specific and nutrition‐sensitive interventions, robust economic evaluations of multi‐sectoral approaches are essential for setting priorities and efficiently allocating resources, particularly in low‐ and middle‐income countries (LMICs) that bear the disproportionate burden of malnutrition. Compared to other global health conditions, such as HIV/AIDS, malaria, other infectious diseases and non‐communicable diseases, the cost‐effectiveness evidence base for nutrition interventions is limited. Notably, a bibliometric review of 614 economic evaluations of health interventions in LMICs found that only 3% pertained to malnutrition and/or anaemia (Pitt et al., 2016). In addition, less than 6% of the Global Health CEA registry, a database of cost‐effectiveness studies evaluating a range of health interventions worldwide, cover interventions to address nutritional deficiencies (Center for Evaluation of Value and Risk in Health [CEVR], 2019). With the growing awareness of the importance of economic evaluation evidence for resource allocation, priority setting, scaling of effective solutions and global and national funding decisions, recently there has been a flurry of systematic reviews that shed light on the costs and benefits of interventions to address nutritional deficiencies in global settings. Two recently published systematic reviews of economic evaluations of interventions provide evidence on preventive nutrition interventions, such as supplementation, infant and young child feeding, therapeutic nutrition interventions (interventions to treat undernutrition and micronutrient deficiencies), fortification and cash transfers linked to improved nutritional outcomes (Njuguna et al., 2020; Ramponi et al., 2020). A third recent study by Das et al. (2020) provides a systematic review of both the effectiveness and cost‐effectiveness of interventions that manage acute malnutrition in children in LMICs. Baek et al. (2021) systematic review of economic evaluations of child nutrition in interventions in LMICs notes a dramatic increase in the number of published economic evaluations of child nutrition interventions between the 2000s and 2010s. Despite this increase, most of the published literature evaluates the cost‐effectiveness of nutrition‐specific interventions.

It may not be coincidental that there are persistent evidence gaps for nutrition‐sensitive interventions and multi‐sectoral approaches. First, it is a substantive challenge to capture and value the diverse benefits associated with multi‐sectoral strategies to improve health outcomes. Multi‐sectoral nutrition strategies produce a wide variety of tangible and intangible benefits to individuals, households and communities. Many nutrition‐sensitive interventions have been shown to significantly improve dietary practices, enhance care practices and reduce the prevalence of disease (Sharma et al., 2021). These interventions lead to improved nutrition and health outcomes through food production, nutrition‐related knowledge, agricultural income and women's empowerment (Sharma et al., 2021). Tangible outcomes can readily be presented in monetary terms and included in economic analysis. These include monetary outcomes like changes in food production, agricultural income and labour productivity. They also include health and nutrition outcomes such as stunting and wasting which have associated morbidity and mortality that can be valued for economic purposes. On the other hand, intangible outcomes, such as women's empowerment, are often measured using methods that are more difficult to value, such as qualitative inquiry or the use of indices.

Health economic evaluation is concerned with the health and monetary benefits resulting from a policy or intervention. Health benefits can be measured with a variety of health and nutrition metrics and can be assigned monetary values. Monetary benefits may refer to averted medical costs or increases in productivity from an intervention. There are three main types of economic evaluation comparing the costs with the consequences of an intervention: cost‐effectiveness analysis (CEA), cost‐utility analysis (CUA) and benefit‐cost analysis (BCA; Drummond et al., 2015). Economic evaluations require distinct considerations when used to evaluate nutrition strategies. CEA compares costs to one specific outcome at a time (such as cost per case of wasting averted) in a cost‐effectiveness ratio. Therefore, these ratios cannot capture the full range of benefits resulting from a multi‐sectoral intervention. CUA calculates costs in terms of health‐adjusted life years such as disability‐ or quality‐adjusted life years (DALYs or QALYs). These measurements express various health‐related outcomes in terms of ‘utility’, an economic concept related to the level of ‘satisfaction’ (or lack thereof) experienced in various health states. Utility‐based metrics can facilitate comparisons between interventions addressing different diseases, and they enable analysts to include multiple health states in one cost‐utility ratio; however, the health benefits included in the evaluation are still limited to death or disability. Finally, a BCA presents all intervention benefits in monetary terms, and therefore each analysis can include a wider range of current and future health and economic benefits. However, valuation of intangible benefits in BCA studies is restricted to the available (and limited) evidence on willingness to pay and revealed preferences for health and nutrition outcomes. Despite the challenges involved in assigning monetary value to intangible impacts and outcomes, for the remainder of this paper, we use the term benefit to refer to all tangible and intangible impacts and outcomes of multi‐sectoral nutrition interventions which have intrinsic value to individuals, households and communities in LMICs. Furthermore, the methodology and assumptions used for determining the monetary value of improved health can vary considerably between studies, and some decision‐makers may object to the concept of translating health to a monetary value (Mills, 2014).

Conventional economic evaluations have typically considered a single health sector whose target is to maximise health or minimise costs. Remme et al. (2017) note that this approach fails to consider that multiple sectors contribute to population and individual health outcomes, and that many of the goods and services produced by the healthcare system have benefits beyond health. Nutrition experts have expressed concerns about using economic evaluation methods (such as cost‐effectiveness) given the heterogeneity of nutrition programmes and the challenge of capturing nutrition benefits, especially when some intervention's primary objectives, such as increased food production, fall outside the health sector domain (Levinson & Herforth, 2013). While other assessment options may exist, based on effectiveness of increasing food production and food security and economic viability, if donors or governments must allocate scarce resources across competing sectoral demands, then improved cost‐effectiveness and benefit‐cost ratios are essential for comparing across investments and are a consideration for decision making. In their absence, it may be difficult to advocate for nutrition as it competes with other government priorities.

Compounding the issue is the difficulty in comparing findings from economic evaluations of multi‐sectoral interventions that use different methodologies. Researchers and practitioners from different disciplines often use distinct terminology to describe comparable analytical approaches. More importantly, interventions—particularly those from different sectors—usually have different objectives and intended proximal and distal outcomes. The heterogeneity of multisectoral programmes and their study designs, the range of benefits measured and valued, and concerns related to quality assessment have all been noted by recent systematic reviews (Baek et al., 2021; Njuguna et al., 2020; Ramponi et al., 2020).

The overall aim of this systematic review is to describe the full range of benefits that have been included or excluded from the current literature on cost‐effectiveness and benefit‐cost of nutrition interventions. We have chosen to focus only on the benefits included in a study or as part of a cost‐effectiveness or benefit‐cost ratio, rather than on the specific intervention costs measured in the evaluations. The specific objectives of this systematic review are to: (1) characterise the types of nutrition‐specific and nutrition‐sensitive interventions included in recent economic evaluations and (2) assess the range of terminology and methodological approaches used to value the nutrition‐related benefits of these interventions. We believe this can help to identify research gaps and improve the quality and design of future studies conducted by interdisciplinary teams of nutritionists, epidemiologists and economists. These findings will inform the design of future economic evaluations of multi‐sectoral nutrition interventions seeking to capture and value the broadest possible range of health and economic benefits.

## 2 METHODS

This systematic review complies with the ‘preferred reporting items for systematic reviews and meta‐analyses’ (PRISMA) checklist for conducting a systematic review (Moher et al., 2009), with the exception of evaluation of bias since the aim of the study was to provide a qualitative assessment of benefits rather than to quantify the magnitude of those benefits. Our study protocol is detailed in Appendix A and summarised below. Given no human subjects were involved in this review, an institutional review board was not needed.

### 2.1 Inclusion criteria

We searched for English‐language, peer‐reviewed, empirical evaluations of nutrition‐related interventions, conducted in one or more LMICs, published from 1 January 2010 to 26 September 2019, and reporting at least one ratio comparing intervention costs and benefits (i.e., cost‐effectiveness ratios for CEAs, cost‐utility ratios for CUAs or benefit‐cost ratios for BCAs; Gillespie & van den Bold, 2017). We used the World Bank criteria to define an LMIC (The World Bank, 2020). Nutrition‐related interventions were defined as activities listed in the *Compendium of Actions for Nutrition*, with some exceptions. Both ‘enabling environment’ actions, including research and national policy actions that would not have an isolated impact on nutrition outcomes, and vertical global health interventions preventing a range of infectious diseases that typically fall outside of nutrition‐specific interventions (such as vaccination and prevention of mother‐to‐child transmission of HIV) were excluded. In addition, some interventions were expanded beyond the *Compendium*, where nutrition experts have explicitly demonstrated their effectiveness for improving maternal or child nutrition (Bhutta et al., 2013; Keats et al., 2021); for instance, all malaria prophylaxis and treatment interventions were included, whereas the *Compendium* only listed intermittent preventive treatment of malaria for pregnant women and distribution of bed nets. Table 1 lists the 78 compendium interventions that we considered. Twenty‐three interventions were from the agriculture/food sector, 27 were from the health sector, 12 were from the water, sanitation and hygiene (WASH) sector, and 16 were from the social protection sector.

**Table 1:** List of nutrition interventions included in the systematic search

Agriculture/food	Health	WASH	Social protection
Animal rearing (homestead and extensive)	Complementary feeding promotion	Access to improved sanitation/latrine construction	Conditional cash transfers (CCTs)
Aquaculture and capture fisheries	Control of household air pollution	Access to improved water	General food distribution in emergency settings
Biodiversity (wild foods and local varieties)	Delayed cord clamping	Community‐based sanitation interventions	Health insurance
Biofortification	Deworming	Environmental hygiene promotion	In‐kind food transfers
Cash cropping	Family planning, delayed age at first pregnancy, & birth spacing	Faecal waste management	Money vouchers for food
Consumer behaviour change communication and education	Malaria prophylaxis and treatment	Food hygiene promotion	Public works programmes
Enhancing digestibility & nutritional value of foods	Management of moderate acute malnutrition	Handwashing education and promotion	School feeding
Food safety and aflatoxin prevention	Management of severe acute malnutrition (wasting)	Household water treatment and safe storage	Skills training and asset transfer
Food storage support	Optimal breastfeeding promotion	Improved source water quality	Social security insurance
Fortification—community	Oral rehydration for diarrhoea	Provision of handwashing supplies	Social transfers (Child support grants & noncontributory pensions)
Home gardening	Paid maternity leave	Provision of safe water under special circumstances (humanitarian emergencies)	Take‐home food rations
Household and extension worker nutrition education/behaviour change communication	Prevention/treatment of nutrition‐related non‐communicable diseases	Sanitation marketing	Unconditional cash transfers (UCTs)
Household food storage	Public provision of complementary food for children		User fee removal (health services)
Improved access to inputs and financing	Supplementation: balanced protein energy		Vouchers for child daycare for children to support infant and young child feeding (IYCF)
Insect farming	Supplementation: calcium		Vouchers for maternal health services
Irrigation	Supplementation: folic acid		Weather‐based insurance for crops and livestock
Labelling regulations	Supplementation: iron/iron folic acid		
Malting, drying, pickling and curing	Supplementation: lipid‐based nutrient		
Marketing regulations	Supplementation: multiple micronutrient		
Mass fortification	Supplementation: omega‐3 fatty acid		
Price policies (taxes and subsidies)	Supplementation: vitamin A		
Promotion of processing for income generation	Supplementation: vitamin D		
Rotation and intercropping	Supplementation: vitamin E		
	Supplementation: vitamin K (neonates)		
	Supplementation: zinc		

Abbreviation: WASH: water, sanitation and hygiene.

We also only included studies that measured at least one nutrition‐related outcome. This allowed us to narrow our focus to studies of interventions that included nutrition as a primary or secondary objective and were designed as such, as opposed to interventions that may have incidentally changed nutritional status. For instance, improving access to fertiliser and other agricultural inputs is a *Compendium* intervention, but if a study evaluating this intervention measured only changes in agricultural yield, it would have been excluded from our review. Nutrition‐related outcomes were defined as the following: improvement in nutritional status; monetary savings from averting a nutritional disorder; food security; dietary diversity; nutritional knowledge, attitudes and/or practices; diarrhoeal incidence; household income; and women's empowerment (Black et al., 2013; Herforth & Harris, 2013).

### 2.2 Search strategy

We searched six databases for studies meeting our inclusion criteria: PubMed, Embase, Web of Science, EconLit, Cinahl and the Cochrane Central Register of Controlled Trials. We developed a list of search terms targeting these criteria. This search strategy included several terms for undernutrition (e.g., acute malnutrition and micronutrient deficiencies), since the vast majority of interventions mentioned in the *Compendium* are intended to address nutritional deficiencies. These terms were then optimised for each database by an information specialist before running the search (the full search strategy is presented in Tables A1a, A1b, A1c, A1d, A1e). Endnote was used to identify duplicate results across different databases (Clarivate, 2020). An additional 24 articles were found through expert consultation and the World Health Organization e‐Library of Evidence for Nutrition Actions (eLENA; World Health Organization, 2020).

### 2.3 Screening and assessment

Two reviewers (Jolene Wun, Christopher Kemp or Devon Bushnell) used the Covidence tool (Covidence) to independently assess each study's title and abstract for inclusion and resolve discrepancies. One reviewer (Jolene Wun or Devon Bushnell) then reviewed the full text of screened studies, and if all inclusion criteria were determined to have been met, the reviewer proceeded to enter key study information in a structured data abstraction form. A second reviewer (Jolene Wun, Christopher Kemp, Chloe Puett or Devon Bushnell) then verified each abstraction and the two resolved any differences through discussion.

Data were abstracted from included studies at two levels. At the study level, the abstraction form contained fields for study details (World Bank region, type of intervention[s] and terminology used to describe benefits). At the economic evaluation ratio (hereafter referred to as *ratio*) level, the abstraction form contained fields for the type of ratio included (CEA, CUA or BCA), and the type of benefit. Benefit categories are defined in Table 2.

**Table 2:** Types of benefits associated with nutrition interventions

Benefit	Description	Example
Nutrition status improved	Averted morbidity and mortality associated with nutrition disorders, their associated DALYs/QALYs, or improvements in anthropometry (i.e., stunting and wasting)	Averted case of vitamin A deficiency, wasting or diarrhoea
Other health status improved	Averted morbidity and mortality associated with any other health improvements or their associated DALYs/QALYs	Averted incidence of malaria and HIV
Monetisation of health status improvements	Monetary valuation of improvements in nutrition and other health status	Value of a statistical life year and other methods to monetise value of life years saved
Productivity gain	Increases in future income earnings due to improvements in nutrition and other health status	Change in projected wage rates
Cognitive/education gain	Gains in school attendance, increases in test performance, cognitive and psychomotor development	Additional years of educational attainment
Cost savings: health system	Averted health (or other social service) provider costs	Reduction in medication costs
Cost savings: beneficiary	Averted direct (out‐of‐pocket) costs and indirect (opportunity) costs	Reduction in health facility fees, medication and travel expenses to and from health facilities
Dietary diversity	Increase in the diversity of food consumed	Improvement in household dietary diversity score
Knowledge/attitude/practice	Improvement in knowledge, attitudes, or practices related to nutrition	Awareness of the importance of exclusive breastfeeding and hygiene
Food security	Improvement in the quantity or quality of food access or consumption	Improvement in household food security score
Income	Increase in household income	Increase in current value of agricultural or livelihoods productivity
Women's empowerment	Increase in women's ability to make important life choices, access opportunities, and improve their economic status and wellbeing	Percentage of women and men who are empowered in key domains related to decision making, control of income and time allocation (Women's Empowerment in Agriculture Index, or WEAI)
Mental/social health	Increase in emotional, social, or psychological wellbeing	Decrease in shame or stress or increase in pride from certain activities (e.g., open defecation, ownership of new technologies)

### 2.4 Analyses

For the studies meeting the inclusion criteria, we calculated the number of studies by sector (food/agriculture, health, WASH, social protection or multi‐sector). We also tabulated the number and percentage of ratios including each benefit type; and for ratios that included a nutrition status improvement, the specific illness averted. All analyses were conducted in Microsoft Excel.

## 3 RESULTS

**Figure 1:**
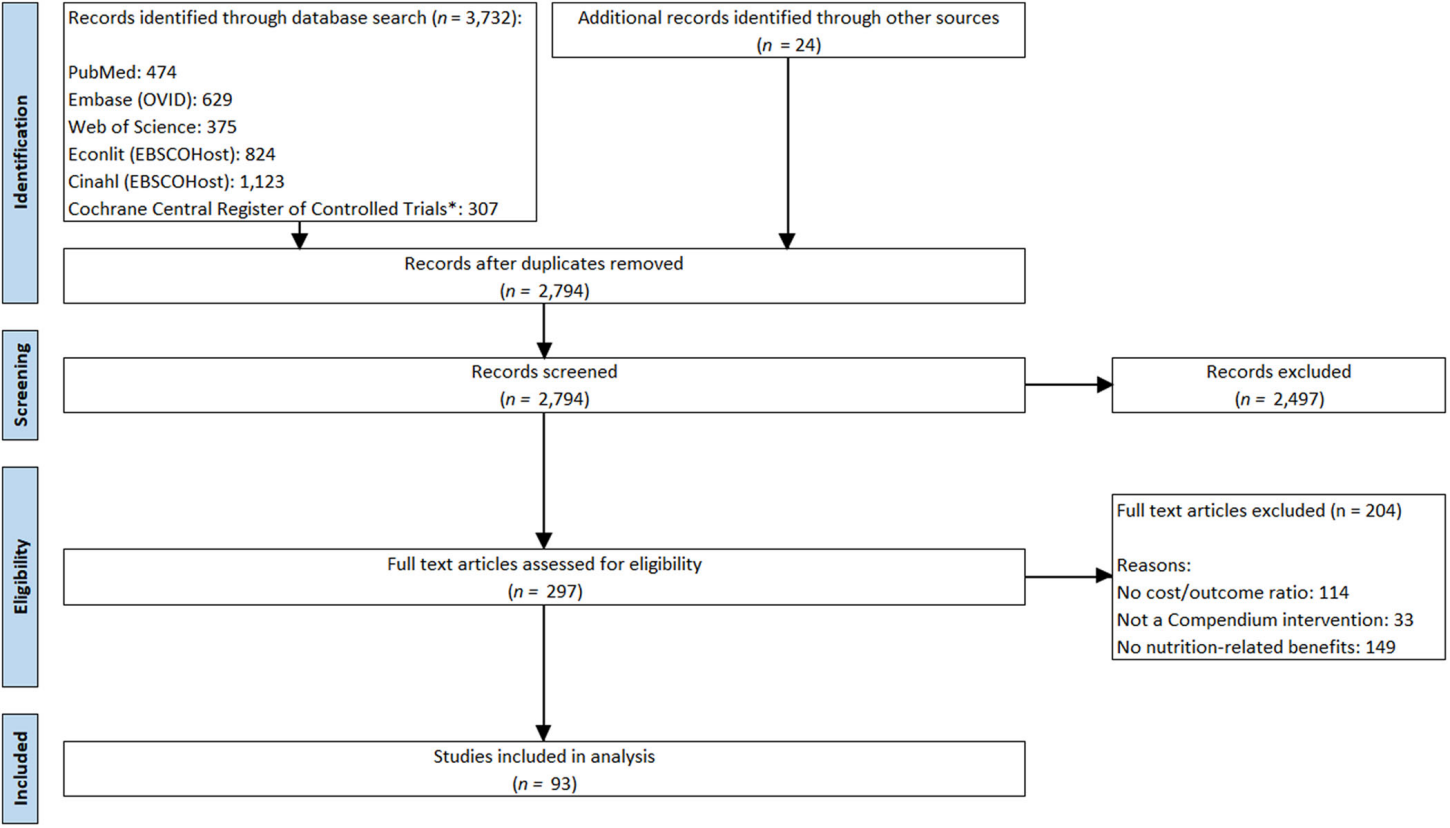
Flow diagram of identified studies

Figure 1 summarises the search results. A total of 2794 studies (24 identified outside of the database search) were screened, and 93 studies met all inclusion criteria. Included studies are summarised in Table A2.

### 3.1 Study‐level descriptive statistics

Studies in our sample covered 44 of the 78 selected *Compendium* nutrition interventions. Table 3 presents a breakdown of specific interventions evaluated. The most commonly assessed interventions were in the health sector: management of severe acute malnutrition/wasting (*n* = 12), zinc supplementation (*n* = 12) and oral rehydration for diarrhoea (*n* = 11). The most commonly assessed interventions in the food/agriculture sector were mass fortification (*n* = 9), and biofortification (*n* = 7). The greatest number of economic evaluations for social protection were for food vouchers (*n* = 4). The greatest number of economic evaluations in the WASH sector were for household water treatment and storage (*n* = 5).

**Table 3:** Nutrition interventions evaluated by studies included in the review, by sector and number of studies

Sector/intervention	Number of studies
**Food/agriculture**	
Mass fortification	9
Biofortification	7
Food/agriculture education or behaviour change communication	3
Improved access to inputs and financing	3
Home gardening	3
Aquaculture and capture fisheries	2
Price policies (taxes and subsidies)	2
Irrigation	2
Cash cropping	1
Food safety and aflatoxin prevention	1
**Health**	
Management of severe acute malnutrition (wasting)	12
Supplementation: zinc	12
Oral rehydration for diarrhoea	11
Supplementation: multiple micronutrient	9
Optimal breastfeeding promotion	9
Supplementation: vitamin A	8
Malaria prophylaxis and treatment	7
Deworming	7
Complementary feeding promotion	7
Management of moderate acute malnutrition	5
Supplementation: balanced protein energy	3
Supplementation: calcium	3
Prevention/treatment of nutrition‐related non‐communicable diseases	1
Supplementation: iron/iron folic acid	1
Supplementation: folic acid	1
**Social protection**	
Money vouchers for food	4
Unconditional cash transfers (UCTs)	3
General food distribution in emergency settings	2
Skills training and asset transfer	2
In‐kind food transfers	2
Health insurance	2
School feeding	1
Take‐home food rations	1
Vouchers for child daycare for children to support infant and young child feeding (IYCF)	1
Conditional cash transfers (CCTs)	1
Public works programmes	1
**WASH**	
Household water treatment and safe storage	5
Access to sanitation/latrine construction	4
Handwashing education and promotion	3
Community‐based sanitation interventions	3
Access to drinking water	2
Improved drinking water quality	1
Environmental hygiene promotion	1
Provision of handwashing supplies	1

Abbreviation: WASH, water, sanitation and hygiene.

Almost half of the studies (41 studies, or 44%) were from Sub‐Saharan Africa; 22% were from South Asia, 15% were from East Asia & the Pacific, 11% from multiple world regions, and the rest in one of the other regions (Europe and Central Asia, Latin America and Caribbean, and Middle East and North Africa). Table 4 summarises the number of studies included in the review by sector, and within each sector, the percentage of studies that conducted each type of economic evaluation and the percentage using specific terms to describe the benefits. Eighty‐one of the included studies (87%) included interventions delivered from only one sector, with almost half from the health sector (43%), followed by food/agriculture (27%), WASH (10%) and social protection (8%). Twelve studies (13%) assessed interventions from more than one sector; however, only eight of them specifically assessed programmes where beneficiaries received interventions from more than one sector (as opposed to comparing interventions from different sectors).

**Table 4:** Included studies by sector, and within each sector, type of economic evaluation conducted and terminology used

*N* (% of all studies)	All (93 [100.0%])	Food/agriculture (25 [26.9%])	Health (40 [43.0%])	Social protection (7 [7.5%])	WASH (9 [9.7%])	Multiple (12 [12.9%])
**Type of economic evaluation**						
Cost‐effectiveness	39 (41.9%)	1 (4.0%)	25 (62.5%)	4 (57.1%)	3 (33.3%)	6 (50.0%)
Cost‐utility	38 (40.9%)	15 (60.0%)	17 (42.5%)	1 (14.3%)	0 (0.0%)	5 (41.7%)
Benefit‐cost	32 (34.4%)	10 (40.0%)	9 (22.5%)	2 (28.6%)	7 (77.8%)	4 (33.3%)
**Terminology used**						
Outcome	87 (68.0%)	15 (55.6%)	38 (70.4%)	15 (93.8%)	6 (42.9%)	13 (76.5%)
Impact	60 (46.9%)	17 (63.0%)	21 (38.9%)	9 (56.3%)	6 (42.9%)	7 (41.2%)
Benefit	72 (56.3%)	21 (77.8%)	28 (51.9%)	2 (12.5%)	8 (57.1%)	13 (76.5%)
Effect	69 (53.9%)	8 (29.6%)	40 (74.1%)	10 (62.5%)	4 (28.6%)	7 (41.2%)
Savings	35 (27.3%)	10 (37.0%)	11 (20.4%)	2 (12.5%)	6 (42.9%)	6 (35.3%)

*Note*: Percentages do not sum to 100% because some studies conducted more than one type of economic evaluation or used more than one term.

Abbreviation: WASH: water, sanitation and hygiene.

Similar proportions of studies conducted CEAs (42%) and CUAs (41%), while slightly fewer studies (34%) conducted BCAs (some studies calculated more than one type of economic evaluation). However, there was considerable variation in the application of each type of economic evaluation across sectors. For example, among WASH studies, BCAs were more common (78%), than CEAs (33%) and no CUAs were conducted. Studies of health interventions most commonly performed CEAs (63%), followed by CUAs (43%). The food and agriculture sector relied primarily on CUAs (60%) and BCAs (40%). Social protection studies mainly were CEAs (57%), followed by BCAs (29%) and CUAs (14%). Eleven studies (12%) conducted more than one type of economic analysis; five of these included both CEAs and CUAs whereby illnesses averted or cured were converted to DALYs or QALYs averted, and six placed a monetary value on either illness or DALYs averted using BCAs.

Sampled studies used a wide range of terminology to refer to intervention benefits, depending on the intervention sector. Across all reviewed studies, the most common term used was ‘benefit’ (63% of studies), followed by ‘outcome’ (62%), and ‘impact’ (52%). However, the majority of studies (80%) used more than one term, with 27% using at least four. Agriculture sector studies most often referred to ‘benefit’ (76%), followed by ‘impact’, then ‘outcome’. Only 32% of agriculture studies referred to ‘effect’ and ‘savings’. Health sector studies primarily referred to ‘effect’ (73%), followed by ‘outcome’, ‘benefit’ and ‘impact’. Only 25% of health sector studies referred to ‘savings’. For social protection studies, the terminology most commonly used was ‘outcome’ (86%), followed by ‘effect’ and ‘impact’ (71% each); ‘benefit’ and ‘savings’ were found in 13% of the studies. WASH studies predominantly referred to ‘benefit’ (78%), followed by ‘impact’ and ‘savings’ (56% each).

Economic evaluation methods appeared to be related to the use of specific terminology. For example, 97% of studies including a BCA ratio referred to ‘benefit,’ while CEAs and CUAs generally referred to ‘outcome’ and ‘effect’. Notably, the term ‘impact’ was used with similar frequency in studies with CUAs (61%) and BCAs (63%) but much less frequently in studies calculating CEAs (33%).

### 3.2 Ratio‐level descriptive statistics

The 93 included studies estimated a total of 128 economic evaluation ratios. Of these ratios, 57 were CEAs (44%), 39 were CUAs (30%) and 32 BCAs (25%). Of the 128 ratios analysed, 76 (59%) measured a single benefit category, 37 (28%) measured two benefit categories, and the remaining 15 (12%) included three or more benefit categories.

Table 5 summarises the types of benefits included in the ratios by sector and type of economic evaluation. The most common benefit was the improvement of nutrition status (56% of all ratios), but this varied by sector: 72% of health sector ratios, 71% of multi‐sector ratios and 56% of food/agriculture ratios included this benefit type; while only 1% of social protection ratios and 7% of WASH ratios did so. Health improvements not related to nutrition, such as malaria and HIV, were included in 9% of the health sector ratios and 24% of multi‐sectoral ratios, but in none of the ratios assessing food/agriculture, social protection, or WASH interventions. WASH ratios, when capturing any benefits resulting from health improvements, instead tended to monetise them (29%), measure the productivity gain resulting from improved health (29%), and/or assess beneficiaries' out‐of‐pocket costs (57%) or indirect costs (such as lost days of work, 42%) from illness, reflecting the high number of BCAs conducted within the sector.

**Table 5:** Types of benefit categories included in economic evaluation ratios (*n* = 128), by intervention sector and evaluation type

		Sector					Economic evaluation type		
Total number of ratios	All (128)	Food/agriculture (27)	Health (54)	Social protection (16)	WASH (14)	Multiple (17)	CEA (57)	CUA (39)	BCA (32)
**Nutrition status improved**	72 (56.3%)	15 (55.6%)	39 (72.2%)	5 (31.3%)	1 (7.1%)	12 (70.6%)	34 (59.6%)	38 (97.4%)	N/A
Anthropometry	12 (9.4%)	0 (0.0%)	6 (11.1%)	2 (12.5%)	0 (0.0%)	4 (23.5%)	12 (21.1%)	N/A	N/A
DALY/QALY	38 (29.7%)	15 (55.6%)	16 (29.6%)	1 (6.3%)	0 (0.0%)	6 (35.3%)	N/A	38 (97.4%)	N/A
Morbidity averted	11 (8.6%)	0 (0.0%)	8 (14.8%)	2 (12.5%)	1 (7.1%)	0 (0.0%)	11 (19.3%)	N/A	N/A
Mortality averted	11 (8.6%)	0 (0.0%)	9 (16.7%)	0 (0.0%)	0 (0.0%)	2 (11.8%)	11 (19.3%)	N/A	N/A
**Other health status improved**	9 (7.0%)	0 (0.0%)	5 (9.3%)	0 (0.0%)	0 (0.0%)	4 (23.5%)	3 (5.3%)	5 (12.8%)	N/A
DALY/QALY	5 (3.9%)	0 (0.0%)	3 (5.6%)	0 (0.0%)	0 (0.0%)	2 (11.8%)	N/A	5 (12.8%)	N/A
Morbidity averted	2 (1.6%)	0 (0.0%)	2 (3.7%)	0 (0.0%)	0 (0.0%)	0 (0.0%)	2 (3.5%)	N/A	N/A
Mortality averted	2 (1.6%)	0 (0.0%)	0 (0.0%)	0 (0.0%)	0 (0.0%)	2 (11.8%)	1 (1.8%)	N/A	N/A
**Monetisation of health status improvements**	10 (7.8%)	3 (11.1%)	2 (3.7%)	0 (0.0%)	4 (28.6%)	1 (5.9%)	N/A	N/A	10 (31.3%)
**Productivity gain**	15 (11.7%)	3 (11.1%)	6 (11.1%)	0 (0.0%)	4 (28.6%)	2 (11.8%)	0 (0.0%)	2 (5.1%)	13 (40.6%)
**Cognitive/education gain**	3 (2.3%)	0 (0.0%)	0 (0.0%)	3 (18.8%)	0 (0.0%)	0 (0.0%)	3 (5.3%)	0 (0.0%)	0 (0.0%)
**Cost savings: health system**	17 (13.3%)	0 (0.0%)	10 (18.5%)	0 (0.0%)	3 (21.4%)	4 (23.5%)	4 (7.0%)	5 (12.8%)	8 (25.0%)
**Cost savings: beneficiary**	28 (21.9%)	1 (3.7%)	15 (27.8%)	0 (0.0%)	8 (57.1%)	4 (23.5%)	7 (12.3%)	6 (15.4%)	15 (46.9%)
Direct (out‐of‐pocket)	23 (18.0%)	1 (3.7%)	11 (20.4%)	0 (0.0%)	8 (57.1%)	3 (17.6%)	6 (10.5%)	4 (10.3%)	13 (40.6%)
Indirect (opportunity cost)	18 (14.1%)	0 (0.0%)	10 (18.5%)	0 (0.0%)	6 (42.9%)	2 (11.8%)	5 (8.8%)	4 (10.3%)	9 (28.1%)
**Dietary diversity**	3 (2.3%)	1 (3.7%)	0 (0.0%)	2 (12.5%)	0 (0.0%)	0 (0.0%)	3 (5.3%)	N/A	0 (0.0%)
**Knowledge/attitude/practice**	9 (7.0%)	0 (0.0%)	3 (5.6%)	0 (0.0%)	6 (42.9%)	0 (0.0%)	9 (15.8%)	N/A	0 (0.0%)
**Food security**	6 (4.7%)	1 (3.7%)	0 (0.0%)	4 (25.0%)	0 (0.0%)	1 (5.9%)	5 (8.8%)	N/A	1 (3.1%)
**Income**	12 (9.4%)	9 (33.3%)	0 (0.0%)	2 (12.5%)	0 (0.0%)	1 (5.9%)	2 (3.5%)	N/A	10 (31.3%)

*Note*: Percentages do not sum to 100% because some ratios included more than one type of benefit. N/A is denoted when it is not possible to include the type of benefit in a certain ratio (health statuses, including anthropometry, morbidity and mortality are measured only as DALYs/QALYs in CUAs and as monetisation of health status in CBAs).

Abbreviations: BCA, benefit‐cost analysis; CEA, cost‐effectiveness analysis; CUA, cost‐utility analysis; DALY, disability‐adjusted life year; QALY, quality‐adjusted life year; WASH, water, sanitation and hygiene.

Benefits other than health status improvements (nutritional and non‐nutritional) also varied by sector. For instance, cognitive or educational gains were included in ratios studying social protection interventions, but not for those evaluating any other sector. Dietary diversity was included in only one agriculture sector ratio, two social protection ratios and none of the other sectors' ratios. Knowledge, attitudes and practices were rarely included in ratios of any sector, with the exception of WASH (6 out of 14 ratios, or 43%); however, all six of these ratios came from one study assessing various attitudes and practices. Increases in household income or assets were included in a higher number of ratios assessing food/agriculture interventions (9 out of 27 ratios, or 33%), compared to 13% of ratios assessing social protection interventions, 6% of evaluations assessing multiple sectors and none of the ratios studying health or WASH interventions.

Notably, only around one‐third of all ratios considered cost savings of any kind. For instance, 47% of BCA ratios included beneficiary cost savings, compared to 12% of CEAs and 13% of CUAs. Cost savings by beneficiaries were more commonly included (22%) than cost savings by the provider (13%), and of those costs, direct costs were more commonly included (18%) as compared to indirect costs (14%).

**Figure 2:**
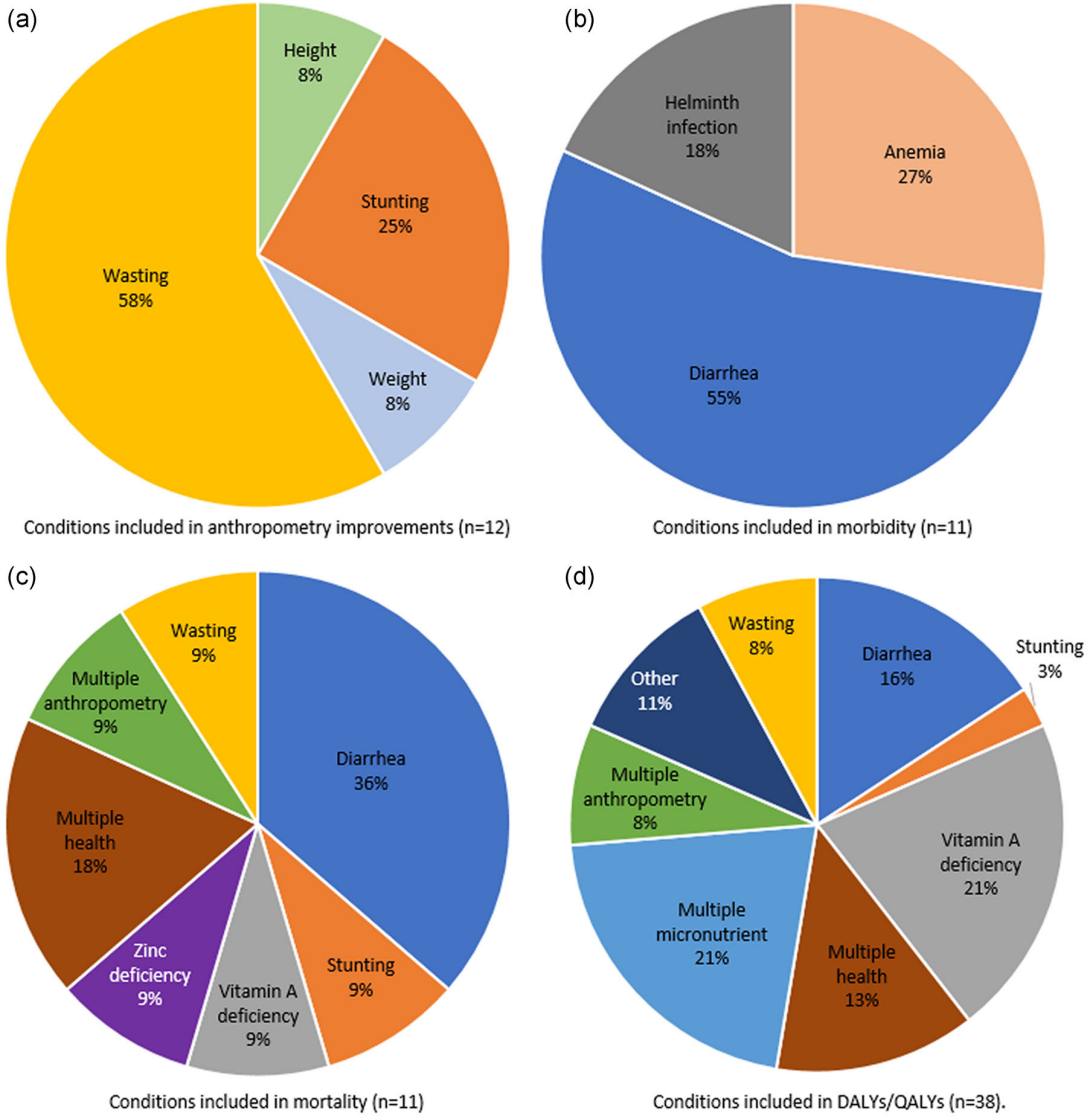
Distribution of conditions included in nutrition‐related improvement in economic evaluation ratios, for four categories of improvements. 'Other' includes iron deficiency anaemia (3%), hepatocellular carcinoma (3%), helminth infection (2%) and folate deficiency (2%)

Within the category of nutritional status improvements, Figure 2 further disaggregates the specific anthropometric, mortality and morbidity benefits measured in CEAs, and nutrition‐related DALYs and QALYs in CUAs. Among CEA ratios incorporating anthropometric assessment of nutrition status improvement, the majority (58%) measured wasting, 25% measured stunting improvements, 9% measured height and 8% measured weight. Among ratios measuring morbidity only, only three health conditions were measured: diarrhoea (55%), anaemia (27%) and helminth infection (18%). Ratios measuring the cost per life saved averted focused on a broader range of benefits, with the top benefit being diarrhoea reduction (36%). Among CUAs including improvements in nutritional status, 42% included more than one type of nutrition disorder averted, with 21% including multiple anthropometric benefits, 21% including multiple micronutrient‐related nutrition disorders and 13% including multiple health‐related disorders. Of those CUA ratios assessing a single nutritional benefit, the most common illnesses averted were vitamin A deficiency (21%), diarrhoea (16%) and wasting (8%).

## 4 DISCUSSION

Our goal was to understand the range of benefits included in the published economic evaluations related to nutrition; how they have been measured and valued, and the terminology used in different sectors to refer to these benefits. To that end, we systematically reviewed economic evaluations of nutrition interventions conducted in LMIC settings. A broad definition of nutrition intervention was used, inclusive of a wide range of nutrition‐specific and nutrition‐sensitive approaches.

Ninety‐three studies were included in the review. The most frequently evaluated interventions were in the health and agriculture sectors. A minority of studies evaluated programmes involving more than one sector. This confirms what other researchers recently found (Ruel et al., 2018): economic evaluations of nutrition interventions are predominantly conducted for nutrition‐specific interventions delivered through the health sector, followed by nutrition‐sensitive interventions delivered through the agricultural sector mostly in the areas of biofortification and fortified foods. There is limited information on the cost‐effectiveness of other agriculture interventions aimed at improving nutrition outcomes. Further, even within the health sector, evidence is missing for several nutrition‐specific interventions, including omega‐3 fatty acids, vitamin D, vitamin E and vitamin K supplementation. There were relatively fewer studies on social protection and WASH interventions, and importantly, very few studies evaluating programmes that implemented interventions from more than one sector, even though multi‐sectoral approaches are important to addressing malnutrition (USAID, 2014). Economic evaluation of more non‐health sector and multipronged interventions are needed to inform decisions on which programmes to implement.

To move towards standard measurement across a range of multi‐sectoral interventions, standardised language around economic evaluations is needed. We assessed the range of terminology and methodological approaches employed to value benefits when compared against intervention costs. We also identified distinctions in terminological use across sectors and study types. There is room for standardising the terminology used in economic evaluations of multi‐sectoral nutrition approaches and interventions. Ideally, terminology could be standardised for use in economic evaluation, depending on the type of analysis to be conducted (CEA, CUA and BCA) and type of benefit that is being assessed (e.g., monetary gain is a benefit, nutrition status improvement is an outcome or effect). In practice, it will take time and coordinated effort to obtain consensus across sectors and disciplines, since the inconsistency across studies reflects differences in both impact evaluation methods and focus across both sectors and disciplines. For example, nutritionists conduct evaluations to measure impact or effectiveness of an intervention, and may focus more on nutritional status, caring practices, educational outcomes, food security or diet‐related changes. Agriculturalists measure impact in crop yields and net incomes, but rarely capture impacts on health or nutrition outcomes. Economists then use the available information on impact or effectiveness results to value the full range of current or future health and economic benefits, converting them to monetary values or utility‐based measurements when feasible.

Economic evaluation of nutrition interventions is challenged by the breadth of outcomes that they affect. In addition to numerous measurable nutrition and health outcomes, there are benefits related to agricultural productivity, income generation, food security, dietary diversity and women's empowerment. This study identifies which benefits have been captured in studies to date and the differences across sectors. The choice of which effects and benefits to include in economic evaluation ratios and the type of economic evaluation selected was found to be strongly related to the sector of the intervention. Health sector and multi‐sectoral evaluations tended to focus on nutritional status improvements and conduct CEAs and CUAs. Other sectors were more mixed in which benefits to include and, with the exception of social protection, conducted BCAs more often.

The choice of benefits and evaluation type may be due to two reasons. First, non‐health sector interventions may be more likely to produce benefits other than health that are important to the intervention (for instance, income generation or time saved in accessing WASH services). For some multi‐sectoral nutrition interventions, BCAs may be the easiest way to capture the full range of benefits. Second, some non‐health interventions may lack the capacity to measure nutrition status. This appears to be the case for several of the social protection interventions, where more proximal benefits such as dietary diversity and food security were measured more frequently than in other sectors. Economic evaluations that lack the capacity, budget and/or time to collect impact data on the final outcomes in the impact pathway for a particular sector could consider including intermediate outcomes related to these omitted final outcomes. In this way, a more complete picture of an intervention's benefits is captured while avoiding double‐counting. For example, one BCA of a national rural drinking water project in India found programme benefits in the categories of health, economy, environment and time savings (Weis et al., 2019). We recommend that authors of economic evaluations (1) clearly define their measures of impact or effectiveness; (2) map out all of the possible benefits arising from the programme; (3) indicate which benefits can be valued as either a health or economic benefit; and (4) clearly describe how the benefit was valued and incorporated into the economic evaluation. We support the recommendation of the Second Panel on Cost‐Effectiveness in Health and Medicine to summarise the full range of benefits (and costs) of interventions in an impact inventory, organised by sector (Sanders et al., 2016).

Comparing the list of potential benefits from multi‐sectoral nutrition interventions (Table 2) with the array of benefits found in this systematic review highlights the predominance of some conditions within the current evidence base—namely wasting, stunting, diarrhoea, anaemia and vitamin A deficiency (as outlined in Figure 2)—and the omission of women's empowerment and mental/social benefits regardless of sector. In addition, cognitive improvements, dietary diversity, food security and changes in knowledge, attitude and practices were measured, but rarely; and cognitive improvements were frequently measured as productivity gains (e.g., higher wages from increased school attendance resulting from improvements in nutrition). These calculations may be highly sensitive to assumptions about future labour markets and economic prospects. Some of the gaps in the existing evidence are due to practical or methodological challenges in benefit measurement, such as placing a monetary value on benefits that do not have a market value. These are intractable challenges that will require future research to advance methods for measurement and quantification.

However, other gaps in counting benefits are easier to address in the shorter‐term. For example, only one‐third of studies included cost savings in terms of averted health care and/or time spent seeking health care. Cost savings can be included in any of the three types of economic evaluation (CEA, CUA or BCA) and should be included more often in economic evaluations for nutrition interventions. Additionally, the majority of economic evaluations in this review (59%) included just one benefit, and about a quarter included two. With the exception of WASH sector evaluations, the inclusion of benefits unrelated to nutrition was relatively rare, so other sectors could consider expanding the range of benefits beyond nutrition and conduct BCAs rather than CUAs or CEAs if their benefits are diverse and can be monetised. There also is scope for more studies to include cognitive, education, or productivity gains associated with investments in nutrition.

This review had several limitations. First, we did not include all *Compendium* interventions. For example, we included malaria treatment and interventions, given their recognised effectiveness in preventing maternal and child nutrition, but excluded other interventions that prevent infant and childhood diseases, notably immunisations and prevention of mother‐to‐child transmission of HIV, which may bias our findings. We also excluded studies that did not explicitly assess a nutrition‐related outcome. We therefore did not evaluate interventions that improve population nutrition incidentally. Second, studies conducted in high‐income countries were excluded, though they may represent a significant proportion of nutrition‐related economic evaluations. This review focused on the unique challenge of implementing and evaluating complex nutrition programmes in low‐resource settings. Third, this review was focused on the science of economic evaluation, and we excluded unpublished and nonpeer‐reviewed studies. Our results may be biased towards investigators from high‐income, English‐speaking settings given barriers to academic publication in English among investigators from lower‐income settings. Finally, our search strategy included explicit search terms for undernutrition and not overnutrition. This review thus may not reflect the full breadth of economic research on strategies to combat nutrition‐related non‐communicable diseases (Nugent et al., 2020).

## CONFLICT OF INTERESTS

The authors declare that there are no conflict of interests.

## AUTHOR CONTRIBUTIONS

CL, CK and JW designed the research study. JW, CK, CP and DB analysed the data. JW, CK, CP, JC and CL wrote the paper.

## Data Availability

The data that support the findings of this study are available from the corresponding author upon reasonable request.

## APPENDIX A SEARCH PROTOCOL

### Specific aims

1. Characterise the benefits measured as part of cost‐effectiveness, cost‐utility, or cost‐benefit analyses in economic evaluations of programmes or interventions aimed at improving nutrition‐related outcomes, including ‘nutrition‐sensitive’ and ‘nutrition‐specific’ interventions.

### Methods

**Inclusion and exclusion criteria**

**Study participants**: Any

**Intervention target population**: Any

**Intervention of interest**: Actions described under the Compendium of Actions for Nutrition, developed by UN Network for Scaling Up Nutrition (SUN)/Renewed Efforts Against Child Hunger and Undernutrition (REACH) Secretariat (see Annex for interventions included).

**Primary outcomes of interest**: Measures of effect/benefit associated with nutrition‐specific/nutrition‐sensitive interventions, including:

- Income
- Dietary diversification
- Food Security
- Women's empowerment
- Knowledge, attitudes and practices related to nutrition
- Diarrhoea
- Malaria
- Nutrition disorders (micronutrient deficiencies, anaemia, stunting, wasting, acute malnutrition and protein‐energy malnutrition)
- Disability‐adjusted life years or mortality associated with nutrition disorders
- Any other benefits/savings from a reduction in a nutrition‐related disease/disorder (including averted treatment costs and improved productivity)

Note: Measures of effect/benefit must be included in the economic analysis (e.g., part of an incremental cost‐effectiveness ratio, benefit‐cost ratio or net benefit estimate).

**Setting**: Any low and middle‐income country (LMIC) per World Bank criteria.

**Type of study**: Any cost‐effectiveness, cost‐utility, or cost‐benefit analysis of any study design (e.g., pre‐post, quasi‐experimental, RCT and modelled).

**Dates**: On or after 1 January 2010

**Languages**: English only

**Types of articles**: Original research articles in peer‐reviewed journals (e.g., no reviews, conference abstracts, book chapters or other grey literature reports).

### Study selection process

**Extract articles**

1. Search each database (see Tables A1a, A1b, A1c, A1d, A1e) and collect articles from subject‐matter experts.
2. Note date of searches, numbers of results from each search and from subject‐matter experts
3. Export results to *Covidence software application* and remove duplicates
4. Note number of results with duplicates removed.
  Review titles/abstracts
5. Two reviewers screen every title/abstract
  a. Screen titles first, hiding abstracts. If title is at all relevant, read abstract.
  b. Criteria for inclusion in the full‐text review include: (1) reference to a SUN compendium intervention; (2) cost‐effectiveness, cost‐utility and/or cost‐benefit analysis; (3) report on at least one effect/benefit/outcome associated with intervention; and (4) reference to LMIC in the title, abstract and/or keywords.
  c. If the reviewer is unsure about whether the article should be included in the full‐text review, he/she will include a comment. These articles will be discussed by the reviewer team, with input from SEEMS UW team as needed, to decide on inclusion in the full‐text review.
  d. Resolve all discrepancies through discussion.
6. Note number of articles included after screening and number excluded.
  Review of full‐text articles and data extraction
7. Two reviewers to screen every full‐text article.
8. Download and read full text of articles included.
  a. Check whether criteria for inclusion are met.
  b. For studies not meeting inclusion/exclusion criteria, note why.
9. Note number of articles included.
10. Develop data abstraction form which will include columns for author, date, setting, type of economic analysis, intervention (sector and type), study design, type of benefit, whether the benefit is compared with cost, beneficiary population, time horizon, whether benefits are modelled or based on empirical data and author terminology.
  a. Test the abstraction form with ~2 articles, revise the form and then test with an additional ~2 articles.
  b. Export to Google Sheets
11. Complete data extraction forms for articles to be included:
  a. Two reviewers will be assigned to each article. One will abstract data for included article on the data abstraction form. The other will validate data abstraction and note any discrepancies.
12. All discrepancies resolved through discussion with the reviewer team.

**Table A1a:** Search strategy for Embase (1996–2019 Week 38)

1	cost effect.mp.
2	cost benefit.mp. or “cost benefit analysis”/
3	cost utility.mp.
4	cost analysis.mp.
5	(“economic evaluation*” or “out of pocket”).mp.
6	1 or 2 or 3 or 4 or 5
7	(“acute malnutrition” or malnutrition or “nutritional care” or “lactating women” or “child nutrition” or “infant nutrition” or “maternal nutrition” or undernutrition or “under‐nutrition” or “severe acute malnutrition” or SAM or CMAM or “community management acute malnutrition” or wasting or wasted or malnourish* or “acutely malnourished” or marasmus).mp.
8	(“diet* diversity” or consumption or intake or food security or “wom* empowerment” or “female empowerment” or diarrhoea or malaria or measles or pneumonia or meningitis or anaemia or anaemia or deficiency or stunt*).mp.
9	(DALY or QALY).mp.
10	child nutrition disorders.mp.
11	infant nutrition disorder.mp.
12	growth disorders.mp. or growth disorder/
13	Protein‐Energy Malnutrition.mp. or protein calorie malnutrition/
14	Kwashiorkor.mp. or kwashiorkor/
15	7 or 8 or 9 or 10 or 11 or 12 or 13 or 14
16	6 and 15
17	(“developing country” or “developing countries” or “low and middle income countries” or LMIC or asia or africa or “south america” or oceania or “latin america” or caribbean).mp.
18	(Afghanistan or Albania or Algeria or Angola or Antigua or Barbuda or Argentina or Armenia or Aruba or Azerbaijan or Bahrain or Bangladesh or Barbados or Benin or Byelarus or Byelorussian or Belarus or Belorussian or Belorussia or Belize or Bhutan or Bolivia or Bosnia or Herzegovina or Hercegovina or Botswana or Brazil or Bulgaria or “Burkina Faso” or “Burkina Fasso” or “Upper Volta” or Burundi or Urundi or Cambodia or “Khmer Republic” or Kampuchea or Cameroon or Cameroons or Cameron or Camerons or “Cape Verde” or “Central African Republic”).mp.
19	(Chad or Chile or China or Colombia or Comoros or “Comoro Islands” or Comores or Mayotte or Congo or Zaire or “Costa Rica” or “Cote d'Ivoire” or “Ivory Coast” or Croatia or Cuba or Cyprus).mp.
20	(Djibouti or “French Somaliland” or Dominica or “Dominican Republic” or “East Timor” or “East Timur” or “Timor Leste” or Ecuador or Egypt or “United Arab Republic” or “El Salvador” or Eritrea or Ethiopia or Fiji or Gabon or “Gabonese Republic” or Gambia or Gaza or Georgia or Georgian or Ghana or “Gold Coast” or Greece or Grenada or Guatemala or Guinea or Guam or Guiana or Guyana or Haiti or Honduras or Hungary or India or Maldives or Indonesia or Iran or Iraq or Jamaica or Jordan or Kazakhstan or Kazakh or Kenya or Kiribati or Korea or Kosovo or Kyrgyzstan or Kirghizia or “Kyrgyz Republic” or Kirghiz or Kirgizstan or “Lao PDR” or Laos or Lebanon or Lesotho or Basutoland or Liberia or Libya or Macedonia or Madagascar or “Malagasy Republic” or Malaysia or Malaya or Malay or Sabah or Sarawak or Malawi or Nyasaland or Mali or Malta or “Marshall Islands” or Mauritania or Mauritius or “Agalega Islands” or Mexico or Micronesia or “Middle East” or Moldova or Moldovia or Moldovian).mp.
21	(Mongolia or Montenegro or Morocco or Ifni or Mozambique or Myanmar or Myanma or Burma or Namibia or Nepal or “Netherlands Antilles” or “New Caledonia” or Nicaragua or Niger or Nigeria or “Northern Mariana Islands” or Oman or Muscat or Pakistan or Palau or Palestine or Panama or Paraguay or Peru or Philippines or Philipines or Phillipines or Phillippines or “Puerto Rico” or Romania or Rumania or Roumania or Rwanda or Ruanda or “Saint Kitts” or “St Kitts” or Nevis or “Saint Lucia” or “St Lucia” or “Saint Vincent” or “St Vincent” or Grenadines or Samoa or “Samoan Islands” or “Navigator Island” or “Navigator Islands”).mp.
22	(“Sao Tome” or “Saudi Arabia” or Senegal or Serbia or Montenegro or Seychelles or “Sierra Leone” or Slovenia or “Sri Lanka” or Ceylon or “Solomon Islands” or Somalia or Sudan or Suriname or Surinam or Swaziland or Syria or Tajikistan or Tadzhikistan or Tadjikistan or Tadzhik or Tanzania or Thailand or Togo or “Togolese Republic” or Tonga or Trinidad or Tobago or Tunisia or Turkey or Turkmenistan or Turkmen or Uganda or Ukraine or Uzbekistan or Uzbek or Vanuatu or “New Hebrides” or Venezuela or Vietnam or “Viet Nam” or “West Bank” or Yemen or Zambia or Zimbabwe).mp.
23	developing country/
24	17 or 18 or 19 or 20 or 21 or 22 or 23
25	16 and 24
26	limit 25 to yr = “2010 ‐Current”
27	diet supplementation/or nutritional supplement*.mp. or nutrition supplement/
28	biofortification/
29	antenatal psychosocial health assessment.mp.
30	(“postpartum depression” or “delayed cord clamping” or “paid maternity leave” or “Preterm massage” or Breastfeed* or “birth control” or “birth spacing” or “delayed pregnancy”).mp.
31	((malaria or deworm* or diarrhoea or “air pollution”) and (control or prevent* or treatment)).mp.
32	(“ready to use therapeutic food” or “ready to use supplementary food” or “plumpy nut” or plumpysoy or imunut or plumpy* or nutributter or FBF or “fortified flour” or “super cereal”).mp.
33	(“kitchen garden” or “community garden” or “home garden” or “school garden” or crops or horticulture or acquaculture).mp.
34	(“food safety” or “aflatoxin prevention” or “cash crops”).mp.
35	(livestock or cattle or poultry or “dairy farm*” or “animal husbandry” or “animal rearing” or fish* or meat or chicken or goat* or cow* or cattle or pig* or sheep* or fish).mp.
36	(Irrigation or biodiversi*).mp.
37	(agricultur* adj2 (educat* or extensi*)).mp.
38	(“crop rotat*” or intercrop* or “insect farm” or “food storage”).mp.
39	((Malt* or dry* or pickl* or cur* or preserv*) adj2 (food or vegetable or fruit)).mp.
40	(Food adj2 (tax or subsid* or regulation or marketing)).mp.
41	((Hygiene or handwash* or “hand wash*”) adj2 (educat* or promot* or communicat* or behaviour)).mp.
42	((“tippy tap” or “tippy‐tap” or tippy tap or soap) adj2 handwash*).mp.
43	(hygiene adj2 (educat* or promot* or communicat*)).mp.
44	(sanitation adj2 (improv* or basic or community)).mp.
45	(“faecal sludge management” or “faecal waste management” or “child faeces disposal”).mp.
46	((sanitation or latrine or toilet) adj2 marketing).mp
47	(“drinking water” adj2 (chlorine or filter or treatment or storage or improv* or safe)).mp.
48	((“source water” or “water supply”) adj2 improv*).mp.
49	(voucher* or “school meal*” or “cash transfer*” or “In‐kind transfer*” or “health insurance”).mp.
50	27 or 28 or 29 or 30 or 31 or 32 or 33 or 34 or 35 or 36 or 37 or 38 or 39 or 40 or 41 or 42 or 43 or 44 or 45 or 46 or 47 or 48 or 49 (1528106)
51	26 and 50

**Table A1b:** Search strategy for Embase (PubMed)

#1	Search “cost effect*” or “cost‐effect*” or “cost benefit” or “cost‐benefit” or “cost utility” or “cost‐utility” or “return on investment” Field: Title/Abstract
#2	Search “Cost‐Benefit Analysis”[Mesh]
#3	Search “cost analysis” or costs or cost or economics or expenditures or “economic evaluation*” or “out of pocket” or expenses Field: Title/Abstract
#4	Search ((#1) OR #2) OR #3
#5	Search (“acute malnutrition” or malnutrition or “nutritional care” or “lactating women” or “child nutrition” or “infant nutrition” or “maternal nutrition” or undernutrition or “under‐nutrition” or “severe acute malnutrition” or SAM or CMAM or “community management acute malnutrition” or wasting or wasted or malnourish* or “acutely malnourished” or marasmus or OR GAM OR MAM OR wasting OR wasted OR malnourish* OR â€œacutely malnourished” OR marasmus or income or “diet* diversity” or consumption or intake or food security or “wom* empowerment” or “female empowerment” or diarrhoea or malaria or measles or pneumonia or meningitis or anaemia or anaemia or deficiency or stunt* Field: Title/Abstract
#6	Search DALY or QALY Field: Title/Abstract
#7	Search “Child Nutrition Disorders”[Mesh]
#8	Search “Infant Nutrition Disorders”[Mesh]
#9	Search “Growth Disorders”[Mesh]
#10	Search “Protein‐Energy Malnutrition”[Mesh]
#11	Search Kwashiorkor Field: Title/Abstract
#12	Search ((((((#11) OR #10) OR #9) OR #8) OR #7) OR #6) OR #5
#13	Search (#12) AND #4
#14	Search “developing country” OR “developing countries” OR “low and middle income countries” OR lmic OR asia OR africa OR “south america” OR oceania OR “latin america” OR caribbean OR afghanistan OR albania OR algeria OR angola OR antigua OR barbuda OR argentina OR armenia OR aruba OR azerbaijan OR bahrain OR bangladesh OR barbados OR benin OR byelarus OR byelorussian OR belarus OR belorussian OR belorussia OR belize OR bhutan OR bolivia OR bosnia OR herzegovina OR hercegovina OR botswana OR brazil OR bulgaria OR “Burkina Faso” OR “Burkina Fasso” OR “Upper Volta” OR burundi OR urundi OR cambodia OR “Khmer Republic” OR kampuchea OR cameroon OR cameroons OR cameron OR cameron OR “Cape Verde” OR “Central African Republic” OR chad OR chile OR china OR colombia OR comoros OR “Comoro Islands” OR comores OR mayotte OR congo OR zaire OR “Costa Rica” OR “Cote d'Ivoire” OR “Ivory Coast” OR croatia OR cuba OR cyprus OR djibouti OR “French Somaliland” OR dominica OR “Dominican Republic” OR “East Timor” OR “East Timur” OR “Timor Leste” OR ecuador OR egypt OR “United Arab Republic” OR “El Salvador” OR eritrea OR ethiopia OR fiji OR gabon OR “Gabonese Republic” OR gambia OR gaza OR georgia OR georgia OR ghana OR “Gold Coast” OR greece OR grenada OR guatemala OR guinea OR guam OR guiana OR guyana OR haiti OR honduras OR hungary OR india OR maldives OR indonesia OR iran OR iraq OR “Isle of Man” OR jamaica OR jordan OR kazakhstan OR kazakh OR kenya OR kiribati OR korea OR kosovo OR kyrgyzstan OR kirghizia OR “Kyrgyz Republic” OR kirghiz OR kyrgyzstan OR “Lao PDR” OR laos OR lebanon OR lesotho OR basutoland OR liberia OR libya OR macedonia OR madagascar OR “Malagasy Republic” OR malaysia OR malaya OR malay OR sabah OR sarawak OR malawi OR nyasaland OR mali OR malta OR “Marshall Islands” OR mauritania OR mauritius OR “Agalega Islands” OR mexico OR micronesia OR “Middle East” OR moldova OR moldova OR moldovan OR mongolia OR montenegro OR morocco OR ifni OR mozambique OR myanmar OR myanmar OR burma OR namibia OR nepal OR “Netherlands Antilles” OR “New Caledonia” OR nicaragua OR niger OR nigeria OR “Northern Mariana Islands” OR oman OR muscat OR pakistan OR palau OR palestine OR panama OR paraguay OR peru OR philippines OR philippines OR philippines OR philippines Field: Title/Abstract
#15	Search “Developing Countries”[Mesh]
#16	Search “Puerto Rico” or Romania or Rumania or Roumania or Rwanda or Ruanda or “Saint Kitts” or “St Kitts” or Nevis or “Saint Lucia” or “St Lucia” or “Saint Vincent” or “St Vincent” or Grenadines or Samoa or “Samoan Islands” or “Navigator Island” or “Navigator Islands” or “Sao Tome” or “Saudi Arabia” or Senegal or Serbia or Montenegro or Seychelles or “Sierra Leone” or Slovenia or “Sri Lanka” or Ceylon or “Solomon Islands” or Somalia or Sudan or Suriname or Surinam or Swaziland or Syria or Tajikistan or Tadzhikistan or Tadjikistan or Tadzhik or Tanzania or Thailand or Togo or “Togolese Republic” or Tonga or Trinidad or Tobago or Tunisia or Turkey or Turkmenistan or Turkmen or Uganda or Ukraine or Uzbekistan or Uzbek or Vanuatu or “New Hebrides” or Venezuela or Vietnam or “Viet Nam” or “West Bank” or Yemen or Zambia or Zimbabwe Field: Title/Abstract
#17	Search ((#16) OR #15) OR #14
#18	Search (#17) AND #13
#19	Search (Protein* or “multiple micronutrient*” or calcium or “folic acid” or iron or vitamin* or zinc or “fatty acid*”) and supplement* Field: Title/Abstract
#20	Search biofortification Field: Title/Abstract
#21	Search ALPHA or “antenatal psychosocial health assessment” or “postpartum depression” or “delayed cord clamping” or “paid maternity leave” or “Preterm massage” or Breastfeed* or “complementary feeding” or “infant feeding” or “home fortification” or “birth control” or “birth spacing” or “delay* pregnancy” Field: Title/Abstract
#22	Search (malaria or deworm* or diarrhoea or “air pollution”) and (control or prevent* or treatment) Field: Title/Abstract
#23	Search “ready to use therapeutic food” OR “ready to use food” OR “ready to use supplementary food” OR “plumpy nut” OR “plumpynut” OR plumpysup OR imunut OR plumpy* OR nutributter OR FBF OR “fortified blended flour” OR “super cereal” Field: Title/Abstract
#24	Search “kitchen garden*or community garden*” or “home garden*” or “school garden*” or crops or horticulture or acquaculture Field: Title/Abstract
#25	Search “food safety” or “aflatoxin prevention” Field: Title/Abstract
#26	Search “cash crop*” Field: Title/Abstract
#27	Search livestock or cattle or poultry or “dairy farm*” or “animal husbandry” or “animal rearing” or fish* or meat or chicken or goat* or cow* or cattle or pig* or sheep* or fish Field: Title/Abstract
#28	Search Irrigation or biodiversi* Field: Title/Abstract
#29	Search agricultur* AND (educat* OR extensi*) Field: Title/Abstract
#30	Search “farm* field school” Field: Title/Abstract
#31	Search “crop rotat*” or intercrop* or “insect farm” or “food storage” Field: Title/Abstract
#32	Search (Malt* or dry* or pickl* or cur* or preserv*) and (food or vegetable or fruit) Field: Title/Abstract
#33	Search Food and (tax or subsid* or regulation or marketing) Field: Title/Abstract
#34	Search (Hygiene or handwash* or “hand wash*”) and (educat* or promot* or communicat* or behavior) Field: Title/Abstract
#35	Search (“tippy tap” OR “tippy‐tap” OR tippy tap OR “soap”) and handwash* Field: Title/Abstract
#36	Search hygiene and (educat* or promot* or communicat*) Field: Title/Abstract
#37	Search sanitation and (improv* or basic or community) Field: Title/Abstract
#38	Search “faecal sludge management” or “faecal waste management” or “child faeces disposal” Field: Title/Abstract
#39	Search (sanitation or latrine or toilet) and marketing Field: Title/Abstract
#40	Search “drinking water” and (chlorine or filter or treatment or storage or improv* or safe) Field: Title/Abstract
#41	Search (“source water” or “water supply”) and improv* Field: Title/Abstract
#42	Search voucher* or “school meal*” or “cash transfer*” or “In‐kind transfer*” or “health insurance” or insurance Field: Title/Abstract
#43	Search ((((((((((((#19 OR #20 OR #21 OR #22 OR #23 OR #24 OR #25 OR #26 OR #27 OR 28) OR #29) OR #30) OR #31) OR #32) OR #33) OR #34) OR #35) OR #36) OR #37) OR #38) OR #39) OR #40 OR #41 OR #42
#44	Search (#18) AND #43
#45	Search (#18) AND #43 Filters: Publication date from 2010/01/01

**Table A1c:** Search strategy for Web of Science. (Indexes = SCI‐EXPANDED, SSCI, A&HCI, CPCI‐S, CPCI‐SSH, BKCI‐S, BKCI‐SSH, ESCI, CCR‐EXPANDED) Refined by: PUBLICATION YEARS: (2019 OR 2011 OR 2018 OR 2010 OR 2017 OR 2016 OR 2015 OR 2014 OR 2013 OR 2012)

1	TOPIC: (“cost effect” or “cost benefit” or “cost benefit analysis”) *OR* **TOPIC**: ((“economic evaluation*” or “out of pocket”))
2	**TOPIC**: ((“acute malnutrition” or malnutrition or “nutritional care” or “lactating women” or “child nutrition” or “infant nutrition” or “maternal nutrition” or undernutrition or “under‐nutrition” or “severe acute malnutrition” or SAM or CMAM or “community management acute malnutrition” or wasting or wasted or malnourish* or “acutely malnourished” or marasmus)) *OR* **TOPIC**: ((“diet* diversity” or consumption or intake or food security or “wom* empowerment” or “female empowerment” or diarrhoea or malaria or measles or pneumonia or meningitis or anaemia or anaemia or deficiency or stunt*)) *OR* **TOPIC**: (DALY or QALY or “nutrition disorder*” or “growth disorder*” or Kwashiorkor)
3	#2 AND #1
4	**TOPIC**: ((“developing country” or “developing countries” or “low and middle income countries” or LMIC or asia or africa or “south america” or oceania or “latin america” or caribbean)) *OR* **TOPIC**: (Afghanistan or Albania or Algeria or Angola or Antigua or Barbuda or Argentina or Armenia or Aruba or Azerbaijan or Bahrain or Bangladesh or Barbados or Benin or Byelarus or Byelorussian or Belarus or Belorussian or Belorussia or Belize or Bhutan or Bolivia or Bosnia or Herzegovina or Hercegovina or Botswana or Brazil or Bulgaria or “Burkina Faso” or “Burkina Fasso” or “Upper Volta” or Burundi or Urundi or Cambodia or “Khmer Republic” or Kampuchea or Cameroon or Cameroons or Cameron or Camerons or “Cape Verde” or “Central African Republic”) *OR* **TOPIC**: (Chad or Chile or China or Colombia or Comoros or “Comoro Islands” or Comores or Mayotte or Congo or Zaire or “Costa Rica” or “Cote d'Ivoire” or “Ivory Coast” or Croatia or Cuba or Cyprus)
5	**TOPIC**: (Djibouti or “French Somaliland” or Dominica or “Dominican Republic” or “East Timor” or “East Timur” or “Timor Leste” or Ecuador or Egypt or “United Arab Republic” or “El Salvador” or Eritrea or Ethiopia or Fiji or Gabon or “Gabonese Republic” or Gambia or Gaza or Georgia or Georgian or Ghana or “Gold Coast” or Greece or Grenada or Guatemala or Guinea or Guam or Guiana or Guyana or Haiti or Honduras or Hungary or India or Maldives or Indonesia or Iran or Iraq or Jamaica or Jordan or Kazakhstan or Kazakh or Kenya or Kiribati or Korea or Kosovo or Kyrgyzstan or Kirghizia or “Kyrgyz Republic” or Kirghiz or Kirgizstan or “Lao PDR” or Laos or Lebanon or Lesotho or Basutoland or Liberia or Libya or Macedonia or Madagascar or “Malagasy Republic” or Malaysia or Malaya or Malay or Sabah or Sarawak or Malawi or Nyasaland or Mali or Malta or “Marshall Islands” or Mauritania or Mauritius or “Agalega Islands” or Mexico or Micronesia or “Middle East” or Moldova or Moldovia or Moldovian) *OR* **TOPIC**: ((Mongolia or Montenegro or Morocco or Ifni or Mozambique or Myanmar or Myanma or Burma or Namibia or Nepal or “Netherlands Antilles” or “New Caledonia” or Nicaragua or Niger or Nigeria or “Northern Mariana Islands” or Oman or Muscat or Pakistan or Palau or Palestine or Panama or Paraguay or Peru or Philippines or Philipines or Phillipines or Phillippines or “Puerto Rico” or Romania or Rumania or Roumania or Rwanda or Ruanda or “Saint Kitts” or “St Kitts” or Nevis or “Saint Lucia” or “St Lucia” or “Saint Vincent” or “St Vincent” or Grenadines or Samoa or “Samoan Islands” or “Navigator Island” or “Navigator Islands”)) *OR* **TOPIC**: ((“Sao Tome” or “Saudi Arabia” or Senegal or Serbia or Montenegro or Seychelles or “Sierra Leone” or Slovenia or “Sri Lanka” or Ceylon or “Solomon Islands” or Somalia or Sudan or Suriname or Surinam or Swaziland or Syria or Tajikistan or Tadzhikistan or Tadjikistan or Tadzhik or Tanzania or Thailand or Togo or “Togolese Republic” or Tonga or Trinidad or Tobago or Tunisia or Turkey or Turkmenistan or Turkmen or Uganda or Ukraine or Uzbekistan or Uzbek or Vanuatu or “New Hebrides” or Venezuela or Vietnam or “Viet Nam” or “West Bank” or Yemen or Zambia or Zimbabwe))
6	#5 OR #4
7	#6 AND #3
8	**TOPIC**: (“diet supplementation” or “nutritional supplement*” or biofortification) *OR* **TOPIC**: (“antenatal psychosocial health assessment” or (“postpartum depression” or “delayed cord clamping” or “paid maternity leave” or “Preterm massage” or Breastfeed* or “birth control” or “birth spacing” or “delayed pregnancy”))
9	**TOPIC**: (((malaria or deworm* or diarrhoea or “air pollution”) and (control or prevent* or treatment))) *OR* **TOPIC**: ((“ready to use therapeutic food” or “ready to use supplementary food” or “plumpy nut” or plumpysoy or imunut or plumpy* or nutributter or FBF or “fortified flour” or “super cereal”).) *OR* **TOPIC**: ((“kitchen garden” or “community garden” or “home garden” or “school garden” or crops or horticulture or acquaculture) or (“food safety” or “aflatoxin prevention” or “cash crops”)
10	**TOPIC**: ((livestock or cattle or poultry or “dairy farm*” or “animal husbandry” or “animal rearing” or fish* or meat or chicken or goat* or cow* or cattle or pig* or sheep* or fish)) *OR* **TOPIC**: (Irrigation or biodiversity) *OR* **TOPIC**: (“agricultural education” or (“crop rotat*” or intercrop* or “insect farm” or “food storage”))
11	**TOPIC**: ((Malt* or dry* or pickl* or cur* or preserv*) and (food or vegetable or fruit)) *OR* **TOPIC**: (Food and (tax or subsid* or regulation or marketing)) *OR* **TOPIC**: (hygiene or handwash* or sanitation or “drinking water” or “water supply”)
12	**TOPIC**: ((voucher* or “school meal*” or “cash transfer*” or “In‐kind transfer*” or “health insurance”))
13	#12 OR #11 OR #10 OR #9 OR #8
14	#13 AND #7

**Table A1d:** Search strategy for Cinahl (EBSCOhost) and EconLIT (EBSCOHost)

1	TX ((“cost effect” or “cost benefit” or “cost benefit analysis”)) OR TX ((“economic evaluation*” or “out of pocket”)) OR TX (expenditure or spending or cost)
2	TX (((“acute malnutrition” or malnutrition or “nutritional care” or “lactating women” or “child nutrition” or “infant nutrition” or “maternal nutrition” or undernutrition or “under‐nutrition” or “severe acute malnutrition” or SAM or CMAM or “community management acute malnutrition” or wasting or wasted or malnourish* or “acutely malnourished” or marasmus))) OR TX (((“diet* diversity” or consumption or intake or food security or “wom* empowerment” or “female empowerment” or diarrhoea or malaria or measles or pneumonia or meningitis or anaemia or anaemia or deficiency or stunt*))) OR TX ((DALY or QALY or “nutrition disorder*” or “growth disorder*” or Kwashiorkor))
3	1 AND 2
4	TX (((“developing country” or “developing countries” or “low and middle income countries” or LMIC or asia or africa or “south america” or oceania or “latin america” or caribbean))) OR TX ((Afghanistan or Albania or Algeria or Angola or Antigua or Barbuda or Argentina or Armenia or Aruba or Azerbaijan or Bahrain or Bangladesh or Barbados or Benin or Byelarus or Byelorussian or Belarus or Belorussian or Belorussia or Belize or Bhutan or Bolivia or Bosnia or Herzegovina or Hercegovina or Botswana or Brazil or Bulgaria or “Burkina Faso” or “Burkina Fasso” or “Upper Volta” or Burundi or Urundi or Cambodia or “Khmer Republic” or Kampuchea or Cameroon or Cameroons or Cameron or Camerons or “Cape Verde” or “Central African Republic”) OR TOPIC: (Chad or Chile or China or Colombia or Comoros or “Comoro Islands” or Comores or Mayotte or Congo or Zaire or “Costa Rica” or “Cote d'Ivoire” or “Ivory Coast” or Croatia or Cuba or Cyprus)) OR TX (((“Sao Tome” or “Saudi Arabia” or Senegal or Serbia or Montenegro or Seychelles or “Sierra Leone” or Slovenia or “Sri Lanka” or Ceylon or “Solomon Islands” or Somalia or Sudan or Suriname or Surinam or Swaziland or Syria or Tajikistan or Tadzhikistan or Tadjikistan or Tadzhik or Tanzania or Thailand or Togo or “Togolese Republic” or Tonga or Trinidad or Tobago or Tunisia or Turkey or Turkmenistan or Turkmen or Uganda or Ukraine or Uzbekistan or Uzbek or Vanuatu or “New Hebrides” or Venezuela or Vietnam or “Viet Nam” or “West Bank” or Yemen or Zambia or Zimbabwe))) OR TX (((Mongolia or Montenegro or Morocco or Ifni or Mozambique or Myanmar or Myanma or Burma or Namibia or Nepal or “Netherlands Antilles” or “New Caledonia” or Nicaragua or Niger or Nigeria or “Northern Mariana Islands” or Oman or Muscat or Pakistan or Palau or Palestine or Panama or Paraguay or Peru or Philippines or Philipines or Phillipines or Phillippines or “Puerto Rico” or Romania or Rumania or Roumania or Rwanda or Ruanda or “Saint Kitts” or “St Kitts” or Nevis or “Saint Lucia” or “St Lucia” or “Saint Vincent” or “St Vincent” or Grenadines or Samoa or “Samoan Islands” or “Navigator Island” or “Navigator Islands”))) OR TX ((Djibouti or “French Somaliland” or Dominica or “Dominican Republic” or “East Timor” or “East Timur” or “Timor Leste” or Ecuador or Egypt or “United Arab Republic” or “El Salvador” or Eritrea or Ethiopia or Fiji or Gabon or “Gabonese Republic” or Gambia or Gaza or Georgia or Georgian or Ghana or “Gold Coast” or Greece or Grenada or Guatemala or Guinea or Guam or Guiana or Guyana or Haiti or Honduras or Hungary or India or Maldives or Indonesia or Iran or Iraq or Jamaica or Jordan or Kazakhstan or Kazakh or Kenya or Kiribati or Korea or Kosovo or Kyrgyzstan or Kirghizia or “Kyrgyz Republic” or Kirghiz or Kirgizstan or “Lao PDR” or Laos or Lebanon or Lesotho or Basutoland or Liberia or Libya or Macedonia or Madagascar or “Malagasy Republic” or Malaysia or Malaya or Malay or Sabah or Sarawak or Malawi or Nyasaland or Mali or Malta or “Marshall Islands” or Mauritania or Mauritius or “Agalega Islands” or Mexico or Micronesia or “Middle East” or Moldova or Moldovia or Moldovian))
5	3 AND 4
6	TX ((“diet supplementation” or “nutritional supplement*” or biofortification)) OR TX ((“antenatal psychosocial health assessment” or (“postpartum depression” or “delayed cord clamping” or “paid maternity leave” or “Preterm massage” or Breastfeed* or “birth control” or “birth spacing” or “delayed pregnancy”))) OR TX (((“kitchen garden” or “community garden” or “home garden” or “school garden” or crops or horticulture or acquaculture) or (“food safety” or “aflatoxin prevention” or “cash crops”))
7	TX (((malaria or deworm* or diarrhoea or “air pollution”) and (control or prevent* or treatment))) OR TOPIC: ((“ready to use therapeutic food” or “ready to use supplementary food” or “plumpy nut” or plumpysoy or imunut or plumpy* or nutributter or FBF or “fortified flour” or “super cereal”
8	TX ((livestock or cattle or poultry or “dairy farm*” or “animal husbandry” or “animal rearing” or fish* or meat or chicken or goat* or cow* or cattle or pig* or sheep* or fish)) OR (Irrigation or biodiversity) OR (“agricultural education” or (“crop rotat*” or intercrop* or “insect farm” or “food storage”))
9	TX ((Malt* or dry* or pickl* or cur* or preserv*) and (food or vegetable or fruit)) OR (Food and (tax or subsid* or regulation or marketing)) OR (hygiene or handwash* or sanitation or “drinking water” or “water supply”)
10	TX ((voucher* or “school meal*” or “cash transfer*” or “In‐kind transfer*” or “health insurance”))
11	6 OR 7 OR 8 OR 9 OR 10
12	5 AND 11

**Table A1e:** Search strategy for Cochrane Central Register of Controlled Trials (Issue 9 of 12, September 2019)

#1	(“cost effect” or “cost benefit” or “cost benefit analysis”) OR (“economic evaluation*” or “out of pocket”) OR (expenditure or spending or costs)
#2	(“acute malnutrition” or malnutrition or “nutritional care” or “lactating women” or “child nutrition” or “infant nutrition” or “maternal nutrition” or undernutrition or “under‐nutrition” or “severe acute malnutrition” or SAM or CMAM or “community management acute malnutrition” or wasting or wasted or malnourish* or “acutely malnourished” or marasmus) OR (“diet* diversity” or consumption or intake or food security or “wom* empowerment” or “female empowerment” or diarrhoea or malaria or measles or pneumonia or meningitis or anaemia or anaemia or deficiency or stunt*) OR (DALY or QALY or “nutrition disorder*” or “growth disorder*” or Kwashiorkor)
#3	#1 and #2 with Publication Year from 2010 to 2019, in Trials
#4	(“developing country” or “developing countries” or “low and middle income countries” or LMIC or asia or africa or “south america” or oceania or “latin america” or caribbean) OR (Afghanistan or Albania or Algeria or Angola or Antigua or Barbuda or Argentina or Armenia or Aruba or Azerbaijan or Bahrain or Bangladesh or Barbados or Benin or Byelarus or Byelorussian or Belarus or Belorussian or Belorussia or Belize or Bhutan or Bolivia or Bosnia or Herzegovina or Hercegovina or Botswana or Brazil or Bulgaria or “Burkina Faso” or “Burkina Fasso” or “Upper Volta” or Burundi or Urundi or Cambodia or “Khmer Republic” or Kampuchea or Cameroon or Cameroons or Cameron or Camerons or “Cape Verde” or “Central African Republic”)
#5	(Chad or Chile or China or Colombia or Comoros or “Comoro Islands” or Comores or Mayotte or Congo or Zaire or “Costa Rica” or “Cote d'Ivoire” or “Ivory Coast” or Croatia or Cuba or Cyprus) OR (“Sao Tome” or “Saudi Arabia” or Senegal or Serbia or Montenegro or Seychelles or “Sierra Leone” or Slovenia or “Sri Lanka” or Ceylon or “Solomon Islands” or Somalia or Sudan or Suriname or Surinam or Swaziland or Syria or Tajikistan or Tadzhikistan or Tadjikistan or Tadzhik or Tanzania or Thailand or Togo or “Togolese Republic” or Tonga or Trinidad or Tobago or Tunisia or Turkey or Turkmenistan or Turkmen or Uganda or Ukraine or Uzbekistan or Uzbek or Vanuatu or “New Hebrides” or Venezuela or Vietnam or “Viet Nam” or “West Bank” or Yemen or Zambia or Zimbabwe)
#6	(Djibouti or “French Somaliland” or Dominica or “Dominican Republic” or “East Timor” or “East Timur” or “Timor Leste” or Ecuador or Egypt or “United Arab Republic” or “El Salvador” or Eritrea or Ethiopia or Fiji or Gabon or “Gabonese Republic” or Gambia or Gaza or Georgia or Georgian or Ghana or “Gold Coast” or Greece or Grenada or Guatemala or Guinea or Guam or Guiana or Guyana or Haiti or Honduras or Hungary or India or Maldives or Indonesia or Iran or Iraq or Jamaica or Jordan or Kazakhstan or Kazakh or Kenya or Kiribati or Korea or Kosovo or Kyrgyzstan or Kirghizia or “Kyrgyz Republic” or Kirghiz or Kirgizstan or “Lao PDR” or Laos or Lebanon or Lesotho or Basutoland or Liberia or Libya or Macedonia or Madagascar or “Malagasy Republic” or Malaysia or Malaya or Malay or Sabah or Sarawak or Malawi or Nyasaland or Mali or Malta or “Marshall Islands” or Mauritania or Mauritius or “Agalega Islands” or Mexico or Micronesia or “Middle East” or Moldova or Moldovia or Moldovian)
#7	(Mongolia or Montenegro or Morocco or Ifni or Mozambique or Myanmar or Myanma or Burma or Namibia or Nepal or “Netherlands Antilles” or “New Caledonia” or Nicaragua or Niger or Nigeria or “Northern Mariana Islands” or Oman or Muscat or Pakistan or Palau or Palestine or Panama or Paraguay or Peru or Philippines or Philipines or Phillipines or Phillippines or “Puerto Rico” or Romania or Rumania or Roumania or Rwanda or Ruanda or “Saint Kitts” or “St Kitts” or Nevis or “Saint Lucia” or “St Lucia” or “Saint Vincent” or “St Vincent” or Grenadines or Samoa or “Samoan Islands” or “Navigator Island” or “Navigator Islands”)
#8	#4 or #5 or #6 or #7
#9	#8 and #3
#10	(“diet supplementation” or “nutritional supplement*” or biofortification) OR (“antenatal psychosocial health assessment” or “postpartum depression” or “delayed cord clamping” or “paid maternity leave” or “Preterm massage” or Breastfeed* or “birth control” or “birth spacing” or “delayed pregnancy”) OR (“kitchen garden” or “community garden” or “home garden” or “school garden” or crops or horticulture or acquaculture) or (“food safety” or “aflatoxin prevention” or “cash crops”)
#11	((malaria or deworm* or diarrhoea or “air pollution”) and (control or prevent* or treatment)) OR (“ready to use therapeutic food” or “ready to use supplementary food” or “plumpy nut” or plumpysoy or imunut or plumpy* or nutributter or FBF or “fortified flour” or “super cereal”)
#12	(livestock or cattle or poultry or “dairy farm*” or “animal husbandry” or “animal rearing” or fish* or meat or chicken or goat* or cow* or cattle or pig* or sheep* or fish) OR (Irrigation or biodiversity OR “agricultural education” or “crop rotat*” or intercrop* or “insect farm” or “food storage”)
#13	((Malt* or dry* or pickl* or cur* or preserv*) and (food or vegetable or fruit))
#14	(Food and (tax or subsid* or regulation or marketing)) OR (hygiene or handwash* or sanitation or “drinking water” or “water supply”)
#15	(voucher* or “school meal*” or “cash transfer*” or “In‐kind transfer*” or “health insurance”)
#16	#10 or #11 or #12 or #13 or #14 or #15
#17	#16 and #9

**Table A2:** Included studies by author name and year

First author, Year	Country	Sector	Intervention(s)	Analysis type(s)	Result terminology	Ratio and benefits included in ratio
Adgo et al. (2013)	Ethiopia	Food/Agriculture	Cash cropping	BCA	Benefit	BCA: Income from increased agricultural productivity
Akram et al. (2016)	Pakistan	Health	Management of SAM, Supplementation: multiple micronutrient	CEA	None	CEA: Recovered wasting cases
Alderman et al. (2017)	Multiple regions/countries	Health	Complementary feeding promotion, Deworming, Management of SAM	BCA	Impact, Benefit	BCA: Increase in income from averted stunting
Ataniyazova et al. (2014)	Uzbekistan	WASH	Handwashing education and promotion, Provision of handwashing supplies	BCA	Benefit	BCA: Increased income from reduction in other illnesses; averted out‐of‐pocket costs from treatment of diarrhoea, helminth infection and other diseases; averted provider costs from treatment of diarrhoea, helminth infection and other diseases; monetary value of time saved caretaking for and recuperating from diarrhoea, helminth infection and other diseases
Banerjee et al. (2015)	Multiple regions/countries	Social Protection	Skills training and asset transfer, Conditional cash transfers (CCTs), Money vouchers for food, Health insurance	BCA	Outcome, Impact, Benefit, Effect	BCA: Value of livestock transfers
Bekchanov et al. (2016)	Multiple Europe & Central Asia	Food/Agriculture	Irrigation	BCA	Outcome, Benefit, Savings	BCA: Income from aquaculture, agriculture, irrigation and other activities; value of hydroelectricity production
Benin et al. (2011)	Uganda	Food/Agriculture	Household and extension worker nutrition ed./BCC	BCA	Outcome, Impact, Benefit	CEA: Averted deaths from vitamin A deficiency CUA: Averted DALYs from vitamin A deficiency
Bergmann et al. (2017)	Malawi, Mozambique	Health	Management of MAM	CUA	Outcome, Impact, Effect	CUA: Averted provider costs from antiretroviral treatment of HIV; averted DALYs from HIV infection; averted DALYs from wasting
Bernal and Fernandez (2013)	Colombia	Multiple	Supplementation: multiple micronutrient, Vouchers for child daycare for children to support infant and young child feeding (IYCF)	BCA	Outcome, Impact, Benefit, Effect	BCA: Increased income from improved socioeconomic skills; increased income from cognitive development; increased income from improved height‐for‐age
Bhutta et al. (2013)	Multiple regions/countries	Multiple	Supplementation: multiple micronutrient, Supplementation: calcium, Supplementation: balanced protein energy, Mass fortification, Optimal breastfeeding promotion, Complementary feeding promotion, Supplementation: Vitamin A, Supplementation: zinc, Management of MAM, Management of SAM	CEA	Effect	CEA: Life years saved from nutritional deficiencies
Bishai et al. (2015)	Myanmar	Health	Oral rehydration for diarrhoea, Supplementation: zinc	CEA, CUA	Outcome, Effect	CEA (1): Monetary value of time saved caretaking for diarrhoea; averted deaths from acute diarrhoea CEA (2): Gain in height CUA: Monetary value of time saved caretaking for diarrhoea; averted DALYs from acute diarrhoea
Boo et al. (2014)	Nicaragua	Health	Supplementation: multiple micronutrient	BCA	Outcome, Impact, Benefit, Effect	BCA: Increased income from cognitive development and increased school attendance associated with anaemia reduction
Brown et al. (2013)	Multiple Sub‐Saharan Africa	Health	Supplementation: zinc	CEA	Outcome, Impact, Benefit, Effect, Savings	CEA (1): Averted deaths from stunting CEA (2): Averted cases of stunting CUA: Averted DALYs from stunting
Brummett et al. (2011)	Cameroon	Food/Agriculture	Aquaculture and capture fisheries, Household and extension worker nutrition ed./BCC	BCA	Impact, Benefit	BCA: Income from aquaculture
Cameron et al. (2011)	South Africa	WASH	Access to improved water	CEA, BCA	Impact, Benefit, Effect, Savings	BCA: Increased income from improved school performance associated with averted diarrhoea; monetary value of time saved from improved access to water; increased income from averted mortality associated with diarrhoea; monetary value of time saved recuperating from diarrhoea; averted out‐of‐pocket costs from diarrhoea treatment CEA: Averted cases of diarrhoea; averted out‐of‐pocket costs from diarrhoea treatment
Casey et al. (2011)	Vietnam	Health	Supplementation: iron/iron folic acid, Deworming	CEA, BCA	Impact, Benefit, Effect	BCA: Increased income due to averted anaemia CEA: Averted cases of anaemia
Cha et al. (2018)	Ghana	WASH	Improved source water quality	BCA	Outcome, Impact, Benefit, Savings	BCA: Monetary value of time saved caretaking for diarrhoea; monetary value of time saved from improved access to water; averted out‐of‐pocket costs from diarrhoea treatment seeking; increased income due to averted diarrhoea; averted provider costs from diarrhoea treatment
Chhagan et al. (2014)	South Africa	Health	Supplementation: zinc, Supplementation: Vitamin A	CEA	Benefit, Effect, Savings	CEA: Averted provider costs from diarrhoea treatment; averted out‐of‐pocket costs from diarrhoea treatment; averted cases of diarrhoea
Chola et al. (2015)	Uganda	Health	Optimal breastfeeding promotion	CEA, CUA	Outcome, Benefit, Effect	CEA: Exclusive or predominant breastfeeding CUA: Averted DALYs from diarrhoea
Chow et al. (2010)	India	Food/Agriculture	Biofortification	CUA	Impact, Benefit	CUA: Averted DALYs from vitamin A deficiency
Clements (2012)	Albania, Nepal, Uganda	Social Protection	Skills training and asset transfer	BCA	Impact	BCA: Value of livestock transfers
Croce et al. (2010)	Cambodia	Health	Deworming	CEA, BCA	Impact, Benefit, Effect	BCA: Increased income due to averted helminth infection; monetary value of time saved recuperating from helminth infection CEA: Averted cases of schistosomiasis
De Neve et al. (2018)	Madagascar	Health	Deworming	CEA, CUA, BCA	Outcome, Impact, Benefit, Effect	BCA: Monetary value of averted DALYs from helminth infection; increased income from increased school attendance associated with averted helminth infection; averted out‐of‐pocket costs from helminth infection treatment CEA: Averted cases of schistosomiasis and hookworm infections CUA: Averted DALYs from helminth infection
De Steur et al. (2012a)	China	Food/Agriculture	Biofortification	CUA	Impact, Benefit, Effect	CUA: Averted DALYs from micronutrient deficiencies
De Steur et al. (2012b)	China	Food/Agriculture	Biofortification	CUA	Outcome, Impact, Benefit, Savings	CUA: Averted DALYs from vitamin A deficiency; averted DALYs from folate deficiency; averted DALYs from zinc deficiency
Dickinson et al. (2015)	India	WASH	Access to improved sanitation/latrine construction	BCA	Benefit	BCA: Increase in future income from averted stunting; monetary value of mortality reduction related to malaria (value of a statistical life); monetary value of time saved from improved access to sanitation facilities; averted out‐of‐pocket costs from diarrhoea treatment
Doocy et al. (2017)	Syria	Social Protection	General food distribution in emergency settings, In‐kind food transfers, Money vouchers for food	CEA	Outcome, Impact, Effect	CEA: Increase in ‘food availability’ (households reporting sufficient food)
Dragojlovic et al. (2020)	Cambodia	Food/Agriculture	Home gardening	BCA	Outcome, Impact, Benefit, Effect	BCA: Increased income due to averted helminth infection; monetary value of time saved recuperating from helminth infection
Escribano Ferrer et al. (2017)	Ghana	Health	Malaria prophylaxis and treatment, Oral rehydration for diarrhoea	CEA	Effect	CEA (1): Cases of malaria and pneumonia appropriately diagnosed and treated CEA (2): Cases of diarrhoea appropriately diagnosed and treated CEA (3): Cases of pneumonia appropriately diagnosed and treated
Fiedler and Afidra (2010)	Uganda	Food/Agriculture	Mass fortification	CUA	Impact, Effect	CEA: Exclusive or predominant breastfeeding
Fiedler et al. (2012)	India	Food/Agriculture	Mass fortification	CUA	Impact	CUA: Averted DALYs from iron deficiency; averted DALYs from vitamin a deficiency; averted DALYs from zinc deficiency
Fiedler et al. (2013)	Zambia	Food/Agriculture	Mass fortification	CUA	Outcome, Impact, Benefit, Savings	CUA: Averted DALYs from iron deficiency; averted DALYs from vitamin a deficiency; averted DALYs from zinc deficiency
Fiedler and Lividini (2014)	Zambia	Multiple	Mass fortification, Biofortification, Supplementation: Vitamin A	CUA	Impact, Benefit, Effect	BCA: Monetary value of averted DALYs from vitamin A deficiency ($1,000 per DALY)
Fiedler et al. (2014)	Zambia	Health	Deworming, Supplementation: Vitamin A	CUA	Outcome, Impact, Savings	CUA: Averted DALYs from vitamin A deficiency
Fiedler and Semakula (2014)	Uganda	Health	Deworming, Supplementation: Vitamin A	CEA, CUA	Impact, Effect	CEA: Averted deaths from vitamin A deficiency CUA: Averted DALYs from vitamin A deficiency
Fiedler et al. (2015)	Bangladesh	Food/Agriculture	Mass fortification	CUA	Outcome, Impact	CEA: Averted treatment seeking costs; monetary value of time saved caretaking for ill child; recovered wasting cases
Fiedler et al. (2016)	Bangladesh	Food/Agriculture	Aquaculture and capture fisheries	CUA	Outcome, Impact, Benefit	CUA: Averted DALYs from vitamin A deficiency
Fink and Heitner (2014)	Zambia	Health	Supplementation: zinc	CUA	Outcome, Impact, Benefit	CUA: Averted DALYs from diarrhoea; averted DALYs from acute lower respiratory illness and malaria
Gebremedhin et al. (2016)	Ethiopia	Health	Oral rehydration for diarrhoea, Supplementation: zinc	CEA	Outcome, Effect	CEA: Adherence to ORS treatment for diarrhoea
Goudet et al. (2018)	India	Health	Management of MAM, Management of SAM	CUA	Outcome	CUA: Averted DALYs from moderate acute malnutrition; averted DALYs from wasting
Hidrobo et al. (2014)	Ecuador	Social Protection	In‐kind food transfers, Unconditional cash transfers (UCTs), Money vouchers for food	CEA	Outcome	CEA (1): Increase in value of food consumption CEA (2): Increase in caloric intake CEA (3): Increase in food consumption score CEA (4): Improvement in household dietary diversity score CEA (5): Improvement in dietary diversity index
Horton et al. (2011)	India	Food/Agriculture	Mass fortification	BCA	Benefit	BCA: Increase in income from averted anaemia
Hutton (2013)	Multiple regions/countries	WASH	Access to improved sanitation/latrine construction, Access to improved water	BCA	Impact, Benefit, Savings	BCA: Monetary value of time saved recuperating from water‐borne disease; monetary value of mortality reduction related to waterborne illness (value of a statistical life); averted treatment seeking costs; averted provider costs from waterborne illnesses; monetary value of time saved from improved access to water and sanitation facilities
Isanaka et al. (2019)	Mali	Health	Management of MAM, Supplementation: Vitamin A, Deworming	CEA, CUA	Outcome, Effect	CEA: Averted deaths from moderate acute malnutrition; averted deaths from wasting CUA: Averted DALYs from wasting; averted DALYs from moderate acute malnutrition
Joy et al. (2017)	Pakistan	Food/Agriculture	Improved access to inputs and financing	BCA	Benefit	BCA: Monetary value of averted DALYs from zinc deficiency (average income per capita)
Kahn et al. (2012)	Kenya	Multiple	Malaria prophylaxis and treatment, Household water treatment and safe storage	CEA	Impact, Benefit, Effect, Savings	CUA: Averted DALYs from diarrhoea; averted DALYs from HIV infection; averted out‐of‐pocket costs from diarrhoea, HIV and malaria treatment
Kern et al. (2013)	Kenya	Multiple	Malaria prophylaxis and treatment, Household water treatment and safe storage	CEA, CUA	Outcome, Impact, Benefit, Savings	CEA: Averted all‐cause mortality; averted provider costs from antiretroviral treatment of HIV progression CUA: Averted DALYs from malaria, HIV infection and other causes; averted DALYs from diarrhoea; averted provider costs from antiretroviral treatment of HIV progression
Kristjansson et al. (2016)	Multiple regions/countries	Social Protection	School feeding	CEA	Outcome, Impact, Effect	CEA (1): Gain in height CEA (2): Gain in weight CEA (3): Gain in cognitive development and math achievement CEA (4): Gain in psychomotor development CEA (5): Increase in school attendance
Lewycka et al. (2013)	Malawi	Health	Optimal breastfeeding promotion	CEA	Outcome	CEA: Averted years of life years lost from all‐cause mortality among mothers and children
Lividini and Fiedler (2015)	Zambia	Food/Agriculture	Biofortification	CUA, BCA	Outcome, Impact, Benefit, Savings	BCA: Monetary value of averted DALYs from vitamin A deficiency ($1,000 per DALY) CUA: Averted DALYs from vitamin A deficiency
Maheu‐Giroux and Castro (2014)	Tanzania	Health	Malaria prophylaxis and treatment	CUA	Outcome, Effect, Savings	CUA: Averted provider costs from malaria diagnosis and treatment; averted DALYs from malaria; monetary value of time saved recuperating from or caretaking for malaria and anaemia
Meenakshi (2010)	Multiple regions/countries	Multiple	Biofortification, Supplementation: multiple micronutrient	CUA	Outcome, Impact, Benefit	CUA: Averted DALYs from vitamin A deficiency; averted DALYs from zinc deficiency; averted DALYs from iron deficiency
Mejia et al. (2015)	Colombia	Health	Supplementation: zinc	CEA	Outcome, Benefit, Effect	CEA: Averted deaths from acute diarrhoea; averted provider costs from diarrhoea treatment
Okafor and Ekwunife (2017)	Nigeria	Health	Oral rehydration for diarrhoea	CUA	Outcome, Benefit	CUA: Averted DALYs from diarrhoea; averted provider costs from diarrhoea treatment
Patel et al. (2013)	India	Health	Supplementation: zinc	CEA	Outcome, Impact, Benefit, Effect, Savings	CEA: Monetary value of time saved caretaking for diarrhoea; averted out‐of‐pocket costs from diarrhoea treatment; averted hours of diarrhoea; averted provider costs from diarrhoea treatment
Peabody et al. (2017)	Philippines	Social Protection	Health insurance	CUA	Outcome, Impact, Benefit, Effect	CUA: Averted DALYs from wasting
Pecenka et al. (2015)	Ethiopia	Health	Oral rehydration for diarrhoea	CEA, BCA	Outcome, Benefit	BCA: Averted out‐of‐pocket costs from diarrhoea treatment and treatment seeking; averted provider costs from diarrhoea treatment CEA: Averted deaths from diarrhoea
Plessow et al. (2016)	India	Food/Agriculture	Price policies (taxes and subsidies)	CUA	Effect, Savings	CUA: Averted DALYs from anaemia; increase in income from averted iron deficiency anaemia
Puett et al. (2013a)	Chad	Social Protection	Take‐home food rations	CEA	Outcome, Effect, Savings	CEA (1): Averted cases of diarrhoea CEA (2): Averted cases of anaemia
Puett et al. (2013b)	Bangladesh	Health	Management of SAM	CEA, CUA	Outcome	CEA: Recovered wasting cases; averted treatment seeking costs; monetary value of time saved caretaking for ill child CUA: Monetary value of time saved caretaking for ill child; averted treatment seeking costs; averted DALYs from wasting
Puett et al. (2014)	Zimbabwe	Food/Agriculture	Home gardening	CEA	Outcome, Benefit, Savings	CEA (1): Increase in food consumption score; income from selling vegetables from community gardens CEA (2): Improvement in household dietary diversity score; income from selling vegetables from community gardens
Qureshy et al. (2013)	Indonesia	Multiple	Complementary feeding promotion, Supplementation: zinc, Oral rehydration for diarrhoea, Supplementation: multiple micronutrient, Community‐based sanitation interventions	BCA	Benefit, Savings	BCA: Averted out‐of‐pocket costs from diarrhoea treatment; increase in income from averted all‐cause mortality; increase in income from averted stunting; increased in income from averted low birth weight and resulting chronic diseases; averted provider costs from diarrhoea treatment
Rautenberg et al. (2017)	Thailand	Health	Oral rehydration for diarrhoea	CUA	Outcome, Effect, Savings	CUA: Gain in QALYs from diarrhoea
Rautenberg et al. (2018)	Malaysia	Health	Oral rehydration for diarrhoea	CUA	Outcome, Impact, Benefit, Effect, Savings	CUA: Averted provider costs from diarrhoea treatment; gain in QALYs from diarrhoea
Reygadas et al. (2018)	Mexico	WASH	Household water treatment and safe storage	CEA	Outcome	CEA (1): Adoption of household water and treatment system CEA (2): Knowledge of household water and treatment system CEA (3): Access of household water and treatment system CEA (4): Operation and consumption of household water and treatment system CEA (5): Exclusive use of household water and treatment system
Rogers et al. (2018)	Mali	Health	Management of SAM	CEA	Outcome, Effect	CEA: Recovered wasting cases
Rogers et al. (2019a)	Pakistan	Multiple	Household water treatment and safe storage, Management of SAM	CEA	Outcome, Benefit, Savings	CEA: Averted cases of stunting
Rogers et al. (2019b)	Pakistan	Health	Management of SAM, Complementary feeding promotion	CEA	Outcome, Effect	CEA: Recovered wasting cases
Schreinemachers et al. (2016)	Bangladesh	Food/Agriculture	Home gardening	CUA	Outcome, Impact, Effect	CUA: Averted DALYs from zinc deficiency; averted DALYs from vitamin a deficiency; averted DALYs from iron deficiency
Schulze et al. (2013)	Thailand	Food/Agriculture	Irrigation	BCA	Benefit, return, profit	BCA: Income from increased agricultural productivity
Shekar et al. (2016)	Multiple Sub‐Saharan Africa	Multiple	Supplementation: multiple micronutrient, Supplementation: calcium, Supplementation: balanced protein energy, Mass fortification, Optimal breastfeeding promotion, Complementary feeding promotion, Supplementation: Vitamin A, Supplementation: zinc, Management of MAM, Management of SAM	CEA, CUA	Outcome, Benefit	CEA (1): Averted deaths from stunting CEA (2): Averted cases of stunting CUA: Averted DALYs from stunting
Shillcutt et al. (2017)	India	Health	Oral rehydration for diarrhoea, Supplementation: zinc	CEA	Outcome, Impact, Benefit, Effect	CEA: Monetary value of time saved recuperating from or caretaking for diarrhoea; averted out‐of‐pocket costs from diarrhoea treatment and treatment seeking; properly treated cases of diarrhoea
Sicuri et al. (2011)	Gabon	Health	Malaria prophylaxis and treatment	CEA	Effect	CEA: Averted cases of anaemia
Siedenburg (2014)	Ethiopia	Multiple	General food distribution in emergency settings, Unconditional cash transfers (UCTs), Public works programmes, Access to improved sanitation/latrine construction, Household and extension worker nutrition ed./BCC, Improved access to inputs and financing	BCA	Outcome, Impact, Benefit	BCA: Averted provider costs from diarrhoea treatment; averted out‐of‐pocket costs from diarrhoea treatment and treatment seeking
Stenberg et al. (2014)	Multiple regions/countries	Health	Supplementation: multiple micronutrient, Supplementation: balanced protein energy, Supplementation: folic acid, Supplementation: calcium, Oral rehydration for diarrhoea, Supplementation: zinc, Supplementation: Vitamin A, Optimal breastfeeding promotion, Complementary feeding promotion, Management of SAM	BCA	Outcome, Impact, Benefit, Effect	BCA: Monetary value of mortality reduction related to malaria (1.5 times GDP per capita)
Suwantika and Postma (2013)	Indonesia	Health	Optimal breastfeeding promotion	CUA	Outcome, Impact, Benefit, Effect	CUA: Averted out‐of‐pocket costs from diarrhoea treatment and treatment seeking; gain in QALYs from diarrhoea; monetary value of time saved recuperating from rotavirus infection
Svefors et al. (2018)	Bangladesh	Health	Supplementation: multiple micronutrient	CUA	Outcome	CUA: Averted DALYs from all‐cause mortality and stunting
Tekeste et al. (2012)	Ethiopia	Health	Management of SAM	CEA	Outcome	CEA: Monetary value of time saved caretaking for ill child; recovered wasting cases; averted treatment seeking costs
Townsend et al. (2017)	India, China	WASH	Handwashing education and promotion	BCA	Benefit, Savings	BCA: Averted out‐of‐pocket costs from diarrhoea treatment; monetary value of averted DALYs from acute respiratory infection (average income per capita); monetary value of averted DALYs from diarrhoea (average income per capita); monetary value of time saved recuperating from or caretaking for diarrhoea or acute respiratory infection
Trenouth et al. (2018)	Pakistan	Multiple	Unconditional cash transfers (UCTs), Money vouchers for food, Optimal breastfeeding promotion, Complementary feeding promotion, Environmental hygiene promotion	CEA, CUA	Outcome, Effect	CEA (1): Averted cases of stunting CEA (2): Averted cases of wasting CUA: Averted DALYs from stunting; averted DALYs from wasting
Verguet et al. (2015)	Ethiopia	Health	Oral rehydration for diarrhoea, Malaria prophylaxis and treatment	CEA	Benefit, Effect	CEA: Averted deaths from diarrhoea
Walters et al. (2016)	Multiple East Asia & Pacific	Health	Optimal breastfeeding promotion	BCA	Benefit, Savings	BCA: Averted out‐of‐pocket costs from diarrhoea and pneumonia treatment; averted provider costs from diarrhoea and pneumonia treatment; increase in income associated with increased breastfeeding
Walters et al. (2019)	Tanzania	Food/Agriculture	Mass fortification	CUA	Effect	CUA: Averted DALYs from vitamin A deficiency
Watkins et al. (2016)	South Africa	Health	Prevention/treatment of nutrition‐related NCDs	BCA	Impact, Benefit, Effect, Savings	BCA: Averted out‐of‐pocket costs from cardiovascular disease treatment; averted provider costs from cardiovascular disease treatment
Weis et al. (2019)	India	WASH	Access to improved sanitation/latrine construction	BCA	Impact, Benefit, Effect, Savings	BCA: Averted out‐of‐pocket costs from water‐borne illness treatment; monetary value of mortality reduction related to waterborne illness (value of a statistical life)
Whittington et al. (2012)	Multiple regions/countries	Multiple	Handwashing education and promotion, Community‐based sanitation interventions, Household water treatment and safe storage, Malaria prophylaxis and treatment	BCA	Outcome, Benefit, Savings	BCA: Monetary value of time saved recuperating from or caretaking for diarrhoea, cholera and malaria; monetary value of time saved from improved access to water and sanitation facilities; averted provider costs from diarrhoea, cholera and malaria; monetary value of averted DALYs from vitamin a deficiency (gdp per capita); averted out‐of‐pocket costs from diarrhoea, cholera and malaria treatment
Wieser et al. (2018)	Pakistan	Food/Agriculture	Price policies (taxes and subsidies)	CUA	Outcome, Benefit, Effect, Savings	CUA: Averted DALYs from vitamin A deficiency; increase in income associated with averted iodine, iron and vitamin A deficiencies; averted DALYs from iodine deficiency; averted out‐of‐pocket costs from micronutrient deficiency treatment; averted DALYs from iron deficiency anaemia
Wilford et al. (2012)	Malawi	Health	Management of SAM	CEA, CUA	Outcome, Effect	CEA: Averted deaths from wasting CUA: Averted DALYs from wasting
Woode et al. (2018)	Ghana	WASH	Community‐based sanitation interventions	CEA	Impact, Effect	CEA: Safe hygiene behavior
Wossen et al. (2017)	Nigeria	Food/Agriculture	Improved access to inputs and financing	BCA	Outcome, Impact, Benefit, Effect	BCA: Income from increased agricultural productivity
Wu and Yang (2014)	Nigeria, Guinea	Food/Agriculture	Food safety and aflatoxin prevention	CUA	Impact, Benefit	CUA: Averted DALYs from hepatocellular carcinoma
Wynn et al. (2017)	South Africa	Health	Optimal breastfeeding promotion	CEA	Outcome, Benefit, Effect, Savings	CEA (1): Averted cases of stunting CEA (2): Exclusive breastfeeding
Zhang et al. (2018)	China	Food/Agriculture	Biofortification	CUA	Outcome, Impact, Benefit, Savings	CUA: Averted DALYs from zinc deficiency; averted DALYs from iron deficiency; averted DALYs from zinc deficiency; averted DALYs from iron deficiency anaemia
